# Effect of the Extraction Methods on the Physicochemical Characteristics of Collagen Derived from Tilapia (*Oreochromis niloticus*) Skin

**DOI:** 10.1021/acsomega.5c02637

**Published:** 2025-08-07

**Authors:** Denise Tiemi Uchida, Adriane do Nascimento Volnistem, Michael Thomas Cook, Marcos Luciano Bruschi

**Affiliations:** † Laboratory of Research and Development of Drug Delivery Systems, Postgraduate Program in Pharmaceutical Sciences, Department of Pharmacy, 42487 State University of Maringa, Colombo Avenue, 5790, K68, Rooms 210-214, Maringa, Paraná 87020-900, Brazil; ‡ Department of Physics, State University of Maringa, Colombo Avenue, 5790, Maringa, Paraná 87020-900, Brazil; § UCL School of Pharmacy, 4919 University College London, 29-39 Brunswick Square, London WC1N 1AX, England, U.K.

## Abstract

Collagen from the skin of *Oreochromis niloticus* (Tilapia; TS) has gained visibility due to its extraction from waste materials from slaughterhouses, reducing environmental impacts, and because it is biodegradable, biocompatible, and nontoxic. The aim of this work was to evaluate the impact of two extraction methods on the physicochemical characteristics of collagen from TS for pharmaceutical and biomedical applications. The obtained TS was analyzed for its proximate composition. Two types of extractions were conducted using a 2^3^ factorial design: acidic extraction (COL) and acidic extraction using pepsin (pCOL). The characterization of collagen was performed using rheological analyses, Raman spectroscopy, differential scanning calorimetry (DSC), thermogravimetric analysis (TGA), X-ray diffraction (XRD), circular dichroism, and scanning electron microscopy (SEM). The acid extraction method was effective in obtaining collagen both with and without pepsin. The proximate composition of the raw material showed results consistent with those of previous studies, highlighting the quality of collagen for use in pharmaceutical formulations. The extraction COL yielded more than pCOL, with both yields being close to 10%. The denaturation temperature of the collagen was above 32 °C, confirmed by DSC, TGA, and circular dichroism analyses, which is favorable for the intended applications. XRD revealed that the supramolecular structure of the type I collagen was preserved. Raman spectroscopy confirmed the presence of hydroxyproline and characteristic collagen amides, with the pCOL6 extraction showing greater stability. Structural differences between COL5 and pCOL6 were observed in the SEM micrographs, suggesting the potential for future applications in the pharmaceutical and biomedical areas.

## Graphical abstract

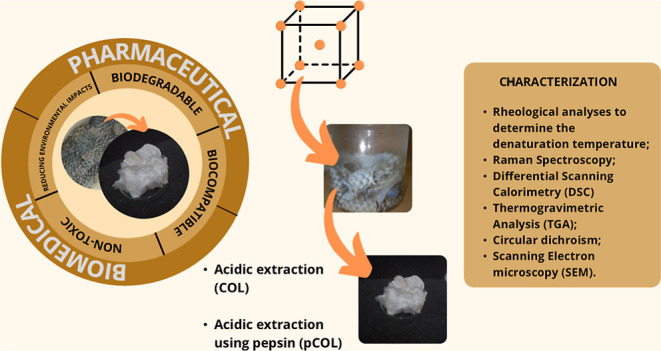

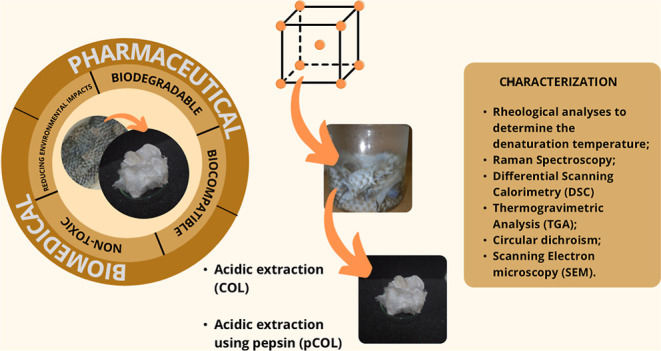

## 1 Introduction

Nowadays, the collagen from Tilapia (*Oreochromis niloticus*) skin (TS) has gained prominence as a sustainable and effective alternative in various fields, particularly in the cosmetics, pharmaceutical, biomaterials, and food industries.^1,2^ Extracted from fish skin, this type of collagen offers advantages over bovine and porcine collagen, as it is more abundant, easier to obtain, and associated with lower risks of zoonotic disease transmission.^3,4^ The use of waste from the fishing industry, such as TS, further contributes to reducing environmental impact, transforming a byproduct into a valuable resource, contributing to sustainability and the circular economy. This versatility, combined with its safety and acceptance by different cultural and religious groups, has driven interest in this biomolecule in various areas of science and technology.

Recent studies have shown that collagen extracted from TS has good biocompatibility, the ability to form fibrils, and potential for biomedical applications such as wound healing and tissue regeneration.^5–8^ In addition, studies have shown that TS possesses antioxidant and anti-inflammatory properties, which enhance its potential in dietary supplements and nutraceutical formulations.^4,9^ Its ability to stimulate collagen production in the human body and improve the health of the skin, hair, and joints positions it as a valuable pharmaceutical ingredient in a wide range of health and wellness products.

Moreover, from a structural perspective, TS collagen is predominantly type I, the most abundant type in the human body, responsible for forming fibers that provide strength and elasticity to tissues.^2,10^ This collagen is easily absorbed by the body, making it ideal for the development of products used in tissue regeneration, wound healing, and aesthetic treatments aimed at improving the skin’s elasticity and firmness. Its ability to form stable gels and its biocompatibility further expand its applications in biomaterials and medical devices, making TS collagen a promising solution in various industries, with enormous potential for growth and innovation.^11,12^

Choosing the most appropriate extraction method for tilapia skin collagen is a decisive factor in ensuring the efficacy and quality of the final material intended for different applications. Several techniques can be used to extract collagen, and the choice of method depends on the origin of the raw material and the type of collagen desired. In general, the process involves three stages: preparation, extraction, and recovery. The preparatory phase includes washing, sanitizing, separating the animal parts, and fragmenting the material, facilitating the following steps. Next, a mild chemical pretreatment is applied—with diluted solutions of acids or bases—to break cross-links in the connective tissue and eliminate noncollagenous components, promoting partial hydrolysis that preserves the collagen structure. Extraction occurs in an aqueous medium, under controlled pH and temperature, using approaches such as acid, enzymatic, or alkaline extraction (which can be seen in Table S1, Supporting Information).^13–31^ More recent methods, such as ultrasound-assisted extraction or extraction with pressurized liquids, have been investigated for their efficiency and shorter processing time. The technique chosen directly impacts the yield, purity, and integrity of the collagen, influencing its potential for application in the pharmaceutical, cosmetic, and biomaterials industries.^10^

However, despite the growing interest in the use of this byproduct, there is still a lack of standardization in extraction protocols, especially regarding the impact of pretreatment conditions on the yield, structural integrity, and thermal stability of collagen. In addition, there are few studies that perform systematic comparisons between these variables using a factorial design approach, which limits the understanding of the synergistic or antagonistic effects of extraction parameters on the final quality of collagen.

Given the growing interest in sustainable biomedical materials and the need to replace traditional sources of collagen, such as bovine and porcine collagen (often associated with contamination risks and religious or cultural restrictions), it is essential to investigate viable, safe, and environmentally friendly alternative sources. In this context, TS, a byproduct largely discarded by the fish processing industry, represents a promising source of collagen, still little explored in terms of optimization of extraction methods aimed at pharmaceutical and biomedical applications. Thus, this study aimed to evaluate the effect of two extraction methods on the physicochemical properties of collagen extracted from tilapia skin, focusing on its potential use in value-added products. For this purpose, a 2^3^ factorial design was used to optimize acid extraction (with and without pepsin), combined with advanced structural and thermal characterization techniques, such as rheological analysis, Raman spectroscopy, circular dichroism, differential scanning calorimetry (DSC), thermogravimetric analysis (TGA), and scanning electron microscopy (SEM). This work contributes not only to the use of agro-industrial waste but also to the development of more efficient and applicable processes on a production scale, with a view to the use of collagen in pharmaceutical formulations and biomaterials.

## 2 Materials and Methods

### 2.1 Sample Material and Chemicals

The waste (TS) discarded in the processing of Tilapia fish (*O. niloticus*) was obtained from a fish slaughterhouse intended for human consumption, located in the city of Umuarama, Parana State, Brazil. The tilapia skins, obtained as unused byproducts of the filleting process, were screened and washed with a 0.9% (w/w) sterile saline solution, and any impurities (such as scales, blood, or residual meat) were removed. After this process, the unused parts were cut into squares (5 × 5 cm) with the help of a knife, stored in plastic packaging, and maintained in a freezer for further analysis.^12^ Sodium hydroxide (NaOH), boric acid, sodium chloride (NaCl), and bromocresol green were purchased from Synth (Sao Paulo, SP, Brazil). Acetic acid (CH_3_COOH) and methyl red were obtained from Vetec (Duque de Caxias, RJ, Brazil), petroleum ether from F.Maia (Sao Paulo, SP, Brazil), sulfuric acid from Quimesp (Guarulhos, SP, Brazil), (4-dimethylaminobenzaldehyde) from Inlab (Sao Paulo, SP, Brazil), and hydrochloric acid (HCl) from Chemycalis (Americana, SP, Brazil). Pepsin from porcine gastric mucosa (≥400 units/mg protein), chloramine T, l-hydroxyproline, and cellulose acetate membranes for dialysis tubing (12,400 Da, 76 mm) were purchased from Sigma-Aldrich (Sao Paulo, SP, Brazil).

### 2.2 Proximal Composition of TS

The following determinations were carried out for characterizing TS: lipid content, protein content (by Kjeldahl method), moisture content (at 110 °C), ash content (incineration at 550 °C), and determination of the amino acid hydroxyproline.^32,33^

The lipid determination was performed by Soxhlet extraction and was performed at least in triplicate. The value in grams of the balloon plus previously desiccated porcelain shards was weighed and noted.^32,33^ Subsequently, 5 g of the previously cleaned TS was weighed on filter paper, deposited in a Soxhlet cartridge, and attached to the balloon. Petroleum ether was added to the cartridge until it covered the siphon. The cooler and heating mantle were turned on. The extraction occurred after 8 h. After the last reflux, the filter paper with the sample was removed, the cooling was turned off, and all the solvent was evaporated. As soon as all the solvent had evaporated, the balloon was kept in an oven at 105 °C for 1 h and cooled in a desiccator to room temperature. The balloon was weighed, and the process of heating in the oven and cooling was repeated until a constant weight was obtained to calculate the lipid content according to eq 1

1 $$lipid\;content=\frac{100\times N}{w}$$

where “*N*” is the weight in grams of lipids and “*w*” is the weight in grams of the sample.

Protein content determination was performed by the modified Kjeldahl method and in triplicate.^32^ On filter paper, 2 g of the sample and 2 g of the catalytic mixture were exactly weighed and then transferred to a Kjeldahl tube, and 10 mL of concentrated sulfuric acid was added. The tube remained on the hot plate at a temperature of 40 °C until the liquid changed from black to translucent. After the solution became translucent, the tube was heated for an additional 30 min and left to cool. In the cooled tube, a volume of 15 mL of distilled water was added, and the material was transferred to a larger digestion tube. The tube was coupled to the distillation set, and 40 mL of a 40% NaOH solution was added. In a 250 mL Erlenmeyer flask, 25 mL of a 4% boric acid solution with three drops of a pH indicator (methyl red) and bromocresol green were added. Distillation occurred until the distillate volume was obtained between 100 and 150 mL, and the solution was titrated with 0.02 N HCl until it turned from green to pink. The titration volume was recorded, and the protein content was calculated according to eqs 2 and 3

2 $$\%nitrogen=\frac{V\times N\times 14\times f}{w}\times 100$$

3 $$\%protein=\%N\times F_{c}$$

where “*V*” is the volume of HCl, “*N*” is the normality of HCl, “14” is the molar mass of nitrogen, “*f*” is the factor of HCl solution, “*w*” is the weight of the sample in mg, “%*N*” is the percentage of nitrogen, and “*F*_c_” is the conversion factor = 5.4.^34–36^

The moisture content analysis consisted of the loss of moisture by desiccation and weighing of the total dry TS of a determined quantity of the sample. To determine the moisture, the crucibles were dried in an oven, and their weight was noted. Approximately 3 g of TS were weighed exactly, and then the crucibles with the sample were taken to an oven at 110 °C for approximately 3 h to completely eliminate moisture. After the determined time, the crucibles containing the samples were taken to the desiccator to cool and then weighed again every 1 h until a constant weight was obtained. The analysis was performed in triplicate, and the results were obtained using the following equation (eq 4)^32^

4 $$moisture\;content\left(\%\right)=\frac{\left(WC+WA\right)-\left(WC+WS\right)}{WA}\times 100$$

where WC is the weight of the crucible, WA is the weight of the sample, and WS is the weight of the dry sample.

The dry residue analysis was performed by determining the difference between 100% and humidity, according to eq 5 ^32^

5 dryresidue(%)=100%−humidityvalue(%)

The analysis of ash content was based on determining the weight of the material subjected to elevated temperature burning and performed in triplicate.^32,33^ The empty porcelain crucible, previously calcined at 550 °C and cooled in a desiccator, was weighed, and the weight was recorded. Approximately 5 g of TS was weighed, recorded, and poured into the crucible. The crucible with the sample was incinerated in a thermal blanket until a mass of carbon was obtained. After this process, the crucible with the sample was placed in a muffle furnace at 550 °C until a white ash was obtained. The muffle furnace was turned off until it reached a temperature of 150 °C, the crucibles were cooled in a desiccator for approximately 25 min, and the weight was recorded. The material was placed in the muffle furnace for 1 h, cooled in a desiccator, and weighed until constant weight, according to eq 6.

6 $$\%ash=\frac{ash\;weight}{sample\;weight}\times 100$$

The hydroxyproline content of the samples was determined spectrophotometrically and in triplicate.^32,33^ The samples were weighed in a glass flask and hydrolyzed in 6 M HCl, at a ratio of 1:10 (w/w), with some glass beads placed at a gentle boil under reflux. Afterward, they were transferred to a volumetric flask, and distilled water was added to complete the volume of 25 mL. This solution was filtered through filter paper and stored at 4 °C in an amber bottle until analysis. Approximately 5 mL of the filtrate was diluted in purified water to a volume of 25 mL, and of this, 2 mL was poured into test tubes, and 1 mL of the chloramine T oxidizing solution was added, where it remained at rest for 20 ± 2 min at a temperature environment. Afterward, 1 mL of the color reagent solution (4-dimethylaminobenzaldehyde) was added, and the tube was closed and shaken vigorously. The tubes were placed in a water bath (60 ± 0.5 °C) for exactly 15 min and then cooled for 3 min in running water. The absorbance was measured against the reagent blank at a wavelength λ = 582 nm. For the standard curve, l-hydroxyproline was used as the standard. The hydroxyproline content was calculated according to eq 7

7 $$H=\frac{\left(A-b\right)}{\left(a\times w\right)}$$

where “*H*” is the hydroxyproline content (g/100 g), “*A*” is the absorbance of the diluted sample filtrate, “*b*” is the linear coefficient of the straight line obtained in the standard curve, “*a*” is the absorptivity, angular coefficient of the straight line obtained in the curve standard, and “*w*” is the sample weight (g).

All analyses were performed at least in three replicate samples. The standard curve for determining the concentration of hydroxyproline is presented in Figure S1 and Table S2 (Supporting Information).

### 2.3 Extraction of Collagen from TS

To define the best method of collagen extraction from TS, a 2^3^-factorial design, with three replicates at the central point, was conducted, totaling 11 extractions (Table 1). The independent variables analyzed were the amounts of NaOH, CH_3_COOH, and mechanical stirring. Two blocks of experiments were performed, with or without pepsin.

**Table 1:** Matrix of Factorial Design 2^3^ (Plus Central Points) for Acidic Extraction of Collagen from Tilapia Skin Using (pCOL) or Not Using Pepsin (COL): Amount of Sodium Hydroxide (NaOH); Amount of Acetic Acid (CH_3_COOH); Mechanical Stirring (ME); with Stirring (wS) for 4 h; 1 h of Stirring and 1 h without Stirring for 4 h (wnS); No Stirring (nS)

Independent variables	Levels		
	–1	0	1
*X*_1_ = NaOH (%)	10	15	20
*X*_2_ = CH_3_COOH (%)	20	35	50
*X*_3_ = ME (type)	nS	wnS	wS

Standard run (extraction)	Levels			(%, w/w)		ME	blocks
	*X*_1_	*X*_2_	*X*_3_	NaOH	CH_3_COOH		
COL1	–1	–1	–1	10	20	nS	COL
COL2	1	–1	–1	20	20	nS	
COL3	–1	1	–1	10	50	nS	
COL4	1	1	–1	20	50	nS	
COL5	–1	–1	1	10	20	wS	
COL6	1	–1	1	20	20	wS	
COL7	–1	1	1	10	50	wS	
COL8	1	1	1	20	50	wS	
COL9 (c)	0	0	0	15	35	wnS	
COL10 (c)	0	0	0	15	35	wnS	
COL11 (c)	0	0	0	15	35	wnS	
pCOL1	–1	–1	–1	10	20	nS	pCOL
pCOL2	1	–1	–1	20	20	nS	
pCOL3	–1	1	–1	10	50	nS	
pCOL4	1	1	–1	20	50	nS	
pCOL5	–1	–1	1	10	20	wS	
pCOL6	1	–1	1	20	20	wS	
pCOL7	–1	1	1	10	50	wS	
pCOL8	1	1	1	20	50	wS	
pCOL9 (c)	0	0	0	15	35	wnS	
pCOL10 (c)	0	0	0	15	35	wnS	
pCOL11 (c)	0	0	0	15	35	wnS	

The dependent variable investigated was the yield of collagen. The extraction yield calculation was conducted based on the relationship between the mass of dry collagen and the wet mass of TS, according to eq 8

8 $$yield\left(\%\right)=\frac{mass\;collagen}{mass\;TS}\times 100$$

The previously cleaned TS were thawed under refrigeration (4 °C) and, prior to weighing, were carefully dried using filter paper to remove excess surface moisture, ensuring consistency in sample handling. Subsequently, the skins were weighed and subjected to pretreatment with NaOH solution (0.1 N; pH 12) under mechanical stirring to remove fat, epithelial cells, and noncollagenous proteins. The samples were stirred for 4 h, changing the solution every 2 h, in a water bath at a temperature up to 10 °C. The TS was washed with cold purified water (temperature less than 10 °C) until neutrality. To extract collagen, a CH_3_COOH solution (0.5 M) was added to TS. Mechanical stirring (350 rpm) was maintained or not for the first 4 h under a bath with a temperature lower than 10 °C. The sample remained refrigerated for 24 h, and after this period, the sample was centrifuged for 40 min at 4000 rpm, and the supernatant was collected. The solution of CH_3_COOH (0.5 M) was added to the supernatant, and the extraction process was carried out two more times. In the third time of extraction, the precipitate was discarded. To purify the extract, the salt precipitation method was conducted with NaCl. The collected supernatant was weighed, and the amount of NaCl to be added was calculated to obtain a 0.9 M NaCl solution. The extract remained under refrigeration overnight, and it was centrifuged for 40 min at 4000 rpm. The supernatant was discarded, and the precipitated extract was resuspended with a 0.5 M CH_3_COOH aqueous solution. For dialysis, the cellulose acetate membrane was hydrated for 24 h, and the extract was added. Afterward, it was dialyzed in a 0.1 M CH_3_COOH aqueous solution for the first 24 h, under refrigeration, and in the following days, it was dialyzed using cold purified water and kept under refrigeration. Finally, the extract was freeze-dried.

Moreover, another extraction was performed using pepsin and the same methodology as previously described. However, the CH_3_COOH aqueous solution (0.5 M) containing 0.1% pepsin was added during extraction to remove telopeptides,^37^ and the extraction procedure was conducted twice more.

All freeze-dried samples of collagen, obtained from the two methods of extraction, pCOL (with pepsin) and COL (without pepsin), were stored in hermetically sealed vials and kept frozen until further analysis.

### 2.4 Characterization of Collagen from TS

#### 2.4.1 Determination of Denaturation Temperature

The denaturation temperature of collagen samples obtained by both COL and pCOL methods was determined by solubilizing them in a 0.5 M CH_3_COOH aqueous solution and monitoring the viscoelastic behavior (oscillatory rheology) of these collagen solutions (1.5 mg/mL) at different temperatures. A controlled gradient and stress rheometer MARS II (Thermo Haake Fisher Scientific Inc., Newington, Germany) was used and equipped with a cone–plate geometry (CP35/2 Ti - 35 mm, separated by 0.052 mm). First, the linear viscoelastic region of each sample (LVR; i.e., the region in which stress was directly proportional to strain, and the storage modulus remained constant) was determined by stress sweep. The temperature sweep analysis was performed at a frequency of 0.1 Hz, and a stress of 0.1 Pa was applied in the range of 10 to 60 °C for 1480 s. At least five replicate samples were analyzed for each collagen sample.

#### 2.4.2 Raman Spectroscopy

Raman spectroscopy analyses were performed using a confocal Raman microscope, model Senterra (Bruker, Germany), with excitation at (λ) 785 nm and a nominal power of 100 mW. Each Raman spectrum was the mean of 30 scans at the same point in the sample. In addition, measurements were carried out at four points in each sample. The excitation laser was focused by a 20× magnification objective, with a spectral resolution of 3–5 cm^–1^, obtained in the range from 400 to 1800 cm^–1^. Raman scattering of samples was detected by using a charged-coupled device (CCD) camera cooled to −84 °C, with an integration time of 10 s for each of the 30 scans. For temperature-related measurements, a temperature control system (T95, Linkam Scientific, U.K.) compatible with a confocal Raman microscope, composed of gas and humidity sensors, was used. The sample was conditioned in a chamber with quartz windows and cooled under liquid nitrogen. The temperature range measured was between 24 and 60 °C. Heating measurements were performed on the sample at a rate of 3 °C/min. The system was stabilized for 10 min with each temperature change. The temperature ramp used for the analysis was 24 °C, 34 °C, 40 °C, 50 °C, and 60 °C. Measurements at room temperature (∼23 °C) were conducted without the temperature chamber. The acquisition of Raman spectra as a function of temperature was obtained using the Linkys 32 program, and the baseline correction and normalization were performed for each spectrum.^38,39^

#### 2.4.3 Thermal Analysis

##### 2.4.3.1 Differential Scanning Calorimetry

The analysis by differential scanning calorimetry (DSC) was performed using an equipment model Q2000 (TA Instruments, New Castle, DE, USA), according to Heng and collaborators (2022), with modifications.^40^ For each analysis, approximately 3 mg of the collagen sample was mixed in purified water at a ratio of 6:1 (water/collagen; w/w), placed in hermetically sealed aluminum pans, and kept refrigerated for 120 min. Afterward, it was subjected to a heating rate of 10 °C/min from 20 to 60 °C, followed by cooling at the same rate, using nitrogen gas at a flow rate of 50 mL/min. Then, the sample underwent a second cycle of heating and cooling, using the same conditions as those in the first cycle.

##### 2.4.3.2 Thermogravimetric Analysis

Thermogravimetric analysis (TGA) was performed using an equipment model Discovery (TA Instruments, New Castle, DE, USA). The analysis was conducted according to Milan and collaborators, with modifications.^41^ TGA was performed by using nitrogen gas at a flow rate of 100 mL/min. For each analysis, 3 mg was weighed in a platinum pan and subjected to a heating rate of 20 °C/min from 10 to 500 °C.

#### 2.4.4 X-ray Diffraction

Structural characterization of the collagen samples was carried out using a Shimadzu XRD-7000 diffractometer with Cu Kα radiation (λ = 1.54439 Å), operating at 40 kV and 30 mA. Data were collected using a θ–2θ configuration, with scanning performed over the 2θ range of 4.0–60.0°, a scan rate of 0.5°/min, and an angular increment (step size) of 0.0104°. The diffraction profiles were analyzed to identify characteristic peaks of collagen, and the interplanar spacings (d-values) were calculated using Bragg’s eq (eq 9)­

9 $$d=\frac{\lambda }{2\sin\;\theta }$$

where λ is the wavelength of the X-ray source (1.54439 Å) and θ is half of the diffraction angle (2θ).

The phases were identified by comparing the obtained diffraction patterns with the standards from the Joint Committee Series of Powder Diffraction Standards (JCPDS) and the database of inorganic crystal structures (ICSD).^42,43^

#### 2.4.5 Analysis by Circular Dichroism

CD spectra were obtained using a Jasco J-1500 spectropolarimeter (Jasco International Co., Tokyo, Japan), connected to a Peltier instrument with temperature control. The collagen samples were prepared at a concentration of 0.1 mg/mL using a solution of 7% glycerol in PBS (pH = 7.3). The spectra were acquired in the 250 – 200 nm region, with a 0.1 cm path length and a quartz cuvette. The [θ]­222 was recorded while heating the samples from 4 to 60 °C at the rate of 0.5 °C/min.^44^

#### 2.4.6 Scanning Electron Microscopy

The surface characteristics of the freeze-dried collagen samples obtained by COL and pCOL (EA and EAp) were analyzed using a scanning electron microscope (Shimadzu SS550, Tokyo, Japan). Samples were deposited on stubs and coated with colloidal gold under an argon atmosphere using a sputter coater.^45^

### 2.5 Statistical Analysis

The effects of NaOH and CH_3_COOH amounts and ME on collagen yield, for both COL and pCOL methods, were statistically evaluated using DoE (Design of Experiment) and compared by analysis of variance (ANOVA). The comparisons of the mean of groups were performed using Tukey’s Honestly significant difference posthoc test. To predict the optimal conditions, a polynomial function was fitted, correlating the relationship between the independent variables and the response according to eq 10

10 $$Y=b_{0}+b_{1}X_{1}+b_{2}X_{2}+b_{3}X_{3}+b_{12}X_{1}X_{2}+b_{13}X_{1}X_{3}+b_{23}X_{2}X_{3}+b_{123}X_{1}X_{2}X_{3}+b_{11}X_{11}^{2}+b_{22}X_{22}^{2}+b_{33}X_{33}^{2}$$

where *Y* is the response as a function of *X*_1_ and *X*_2_; *b*_0_ is a constant term; *b*_1_, *b*_2_, and *b*_3_ are the estimated coefficients of linear terms, and *b*_12_–*b*_33_ are the coefficients of the interaction effect. The polynomial terms (from *X* ^2^_11_ to *X* ^2^_33_) consider the nonlinearity, and the interaction terms (from *X*_1_ *X*_2_ to *X*_3_ *X*_3_) show as the response changes when two factors are simultaneously changed.

The center points were conducted to estimate the pure error variance and the reproducibility of the method. The experiments were randomized to minimize the influence of unexplained variability in responses. Residual analysis was also conducted to validate the assumptions utilized in the analysis of variance and to identify outliers. The proportion of variance explained by the models obtained was given by the multiple and adjusted determination coefficients (*R* ^2^ and *R* ^2^_adj_), whereas the adequacy of the model was determined by a lack-of-fit test. Statistica software version 8.0 (StatSoft Company, Tulsa, OK, USA) was utilized, and in all cases, a level of *p* < 0.05 was accepted to denote significance.

## 3 Results and Discussion

### 3.1 Proximal Composition of TS

It is known that the functional properties of collagen depend on processing parameters (e.g., pH, temperature, and time), pretreatment, and conservation of the raw material. The proximal characterization of the raw material is essential to obtaining the functional properties of collagen. Characterization was carried out with previously cleaned TS (Table 2).

**Table 2:** Physicochemical Characterization of Tilapia Skin (TS) in Nature^a^

Sample	Content (%, w/w)					
	Lipid	Protein	Moisture	Dryness residue	Total ashes	Hydroxyproline
A1	6.13	24.62	67.23	32.77	0.42	1.74
A2	6.21	24.36	67.82	32.18	0.41	1.75
A3	5.87	26.72	68.01	31.99	0.44	1.75
Mean	6.07	25.23	67.69	32.31	0.42	1.74
SD	0.18	1.3	0.41	0.41	0.015	0.01
RSD (%)	2.97	5.15	0.61	1.26	3.57	0.49

a A = sample, SD = standard deviation, RSD = relative standard deviation.

Compared to other studies, the lipid content for TS is between 2 and 15%, the protein content is between 17 and 22%, the humidity is between 64 and 80%, and the ash content is between 0.5 and 12%.^2,10,46,47^ However, it should not be overlooked that comparing literature data is difficult due to the lack of information on fish age and breeding conditions. Although similar, this subtle difference among levels is influenced according to the type of food, climate, sex, age, and species of tilapia used for collagen extraction.^47–50^ Diet directly influences the content of lipids and structural proteins present in the skin, impacting the purity and recovery of collagen. Climate, especially water temperature, alters fish metabolism and can modify the thermal stability of the synthesized collagen. Sexual differences are also relevant since hormones such as estrogen and testosterone modulate the expression of extracellular matrix proteins, including collagen. The age of the fish is one of the most critical factors: older fish have greater intermolecular cross-linking in collagen fibers, forming more stable structures that are less soluble in acidic solutions, which reduces their extraction by conventional methods. In addition, distinct species of tilapia have genetic differences that affect both the composition and the proportion of the present types of collagens. Moisture and ash contents are directly proportional to age, mainly ash due to greater calcification of scales according to the age of the fish.^34,48^

When the amount of fat in TS is high, it is essential that treatments related to its extraction are efficient in completely removing the lipid material.^46^ The presence of lipids in the skin can directly influence the physical and chemical properties of collagen since high lipid levels tend to reduce its solubility in aqueous media. This lower solubility makes it difficult to efficiently separate collagen from other components of the dermal matrix, negatively impacting both the purity and the quality of the extracted material. In addition, high lipid content can compromise the yield of the extraction process, making subsequent steps difficult, such as filtration and recovery of collagen from the solution. Furthermore, when there is the presence of high values of lipids, they undergo oxidation reactions which contribute to the development of undesirable taste and odor.^51,52^ The low lipid content found in this investigation is in accordance with the results found in other studies on TS. The presence of this low lipid content in the sample was expected, as it acts to reduce permeability to water vapor, a property with high values in protein-based films.^53^

Although there is no official standard established to qualify TS regarding its suitability for collagen extraction, knowledge of its proximal composition is essential to define quality parameters and select appropriate extraction methodologies. The composition of the skin directly impacts the efficiency of the process since, for example, high lipid contents may require additional degreasing steps to ensure greater purity of the collagen obtained. Thus, the evaluation of the proximal composition of TS allows the adaptation of the methods to the characteristics of the material. Based on values reported in the literature for skins successfully used in this type of extraction, it was found that the skin studied presented adequate contents, especially regarding the low lipid content and high protein fraction. Therefore, collagen extraction was carried out using acetic acid, with and without the presence of pepsin, resulting in the samples COL and pCOL, respectively.

### 3.2 Experimental Design for the Optimization of Collagen Extraction from TS

Studies have demonstrated that the TS collagen extraction method using CH_3_COOH results in a higher yield, due to its small molecular size and lower ionization constant, providing more efficient swelling of the collagen before conversion into gelatin.^54^ For this reason, CH_3_COOH was selected as the collagen-extracting liquid. The yield contents of collagen samples are described in Table 3.

**Table 3:** Yield Values and Hydroxyproline Content of the Extracts Obtained by the Methodology Using Acetic Acid with or without Pepsin (Acid Extraction and Acid Extraction with Pepsin), Using the 2^3^-Factorial Design with Three Replications at the Central Point

Extraction	Content (%, w/w)			
	Acid extraction (COL)		Acid extraction with pepsin (pCOL)	
	Yield	Hydroxyproline	Yield	Hydroxyproline
1	3.15	25.75 ± 0.02	4.52	2.76 ± 0.04
2	0.45	1.62 ± 0.02	4.51	2.37 ± 0.01
3	2.92	1.69 ± 0.07	6.85	3.34 ± 0.07
4	0.72	11.91 ± 0.07	2.74	4.07 ± 0.07
5	11.50	10.44 ± 0.37	4.23	3.50 ± 0.02
6	8.19	8.71 ± 0.06	7.80	3.46 ± 0.01
7	1.17	1.76 ± 0.05	3.23	2.76 ± 0.03
8	2.67	8.39 ± 0.09	4.13	3.07 ± 0.03
9	5.76	1.92 ± 0.02	2.98	4.02 ± 0.05
10	4.67	1.78 ± 0.02	4.26	3.69 ± 0.16
11	3.96	3.11 ± 0.05	3.62	2.93 ± 0.03

For the yield using the COL method, the mathematical model that best fitted the experimental results was the linear model. With an adjusted coefficient of determination (*R* ^2^adj) of 0.89 and a nonsignificant lack of adjustment (p = 0.26), the model was used to predict values (*y* = 4.10–3.95*X*_2_ + 4.07*X*_3_ – 3.97*X*_2_ *X*_3_). The values of the equation coefficients and the respective *p*-values are presented in Table S3 (Supporting Information). It was observed that the amount of NaOH (*X*_1_) and CH_3_COOH (*X*_2_) displayed a significant negative influence on the yield performance (*p* < 0.05). As they increase, the yield decreases. On the other hand, as the mechanical stirring (*X*_3_) increased, the yield also increased and indicating a positive influence (*p* < 0.05). The term *X*_2_ *X*_3_ showed a significant effect (*p* = 0.0144) with a negative influence on performance, indicating that the combined influence of the two factors on performance is attenuated when compared to the expectation based on individual effects. This negative two-term interaction indicates that the joint effect of these two factors is smaller than the sum of the individual effects of each factor alone.

The effect of the independent variables on the acid extraction yield is displayed in Figure 1. Note that the response surface graph shows a reddish color at the points where the variables resulted in higher yield. *X*_1_ and *X*_2_ increased the yield at the smallest volumes (Figure 1A). In Figure 1B,C, the yield increases when mechanical agitation is applied, while the volume used during the NaOH and CH_3_COOH extraction processes is smaller, respectively.

**Figure 1:**
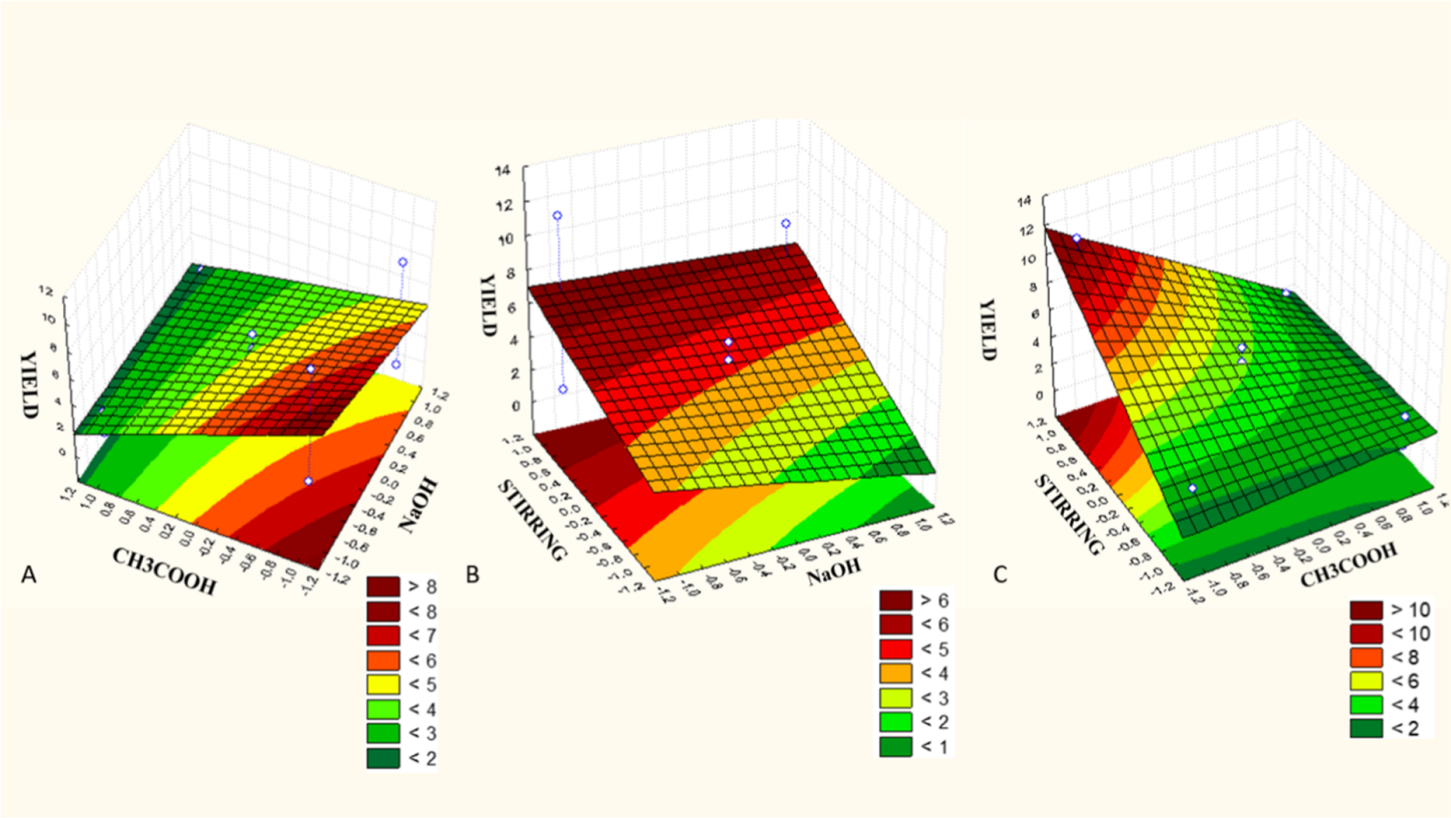
Yield response surface graphs from acid extraction: (A) Response surface of NaOH (sodium hydroxide—*X*_1_) and CH_3_COOH (acetic acid—*X*_2_); (B) response surface between mechanical stirring (*X*_3_) and sodium hydroxide (*X*_1_); (C) response surface between mechanical stirring (*X*_3_) and acetic acid (*X*_2_).

For pCOL, the model that best represents the highest extraction yield was the model represented by the equation (*y* = 4.44 + 1.08*X*_1_ *X*_3_). The values of the equation coefficients and the respective *p* values are presented in Table S4 (Supporting Information). It is noted that the *X*_1_ *X*_3_ interaction presented a positive response in yield. In other words, when the interaction is positive, the combined influence of the two factors is amplified, indicating a synergy between them. This indicates that the combination of higher levels of both factors is associated with a significantly larger effect on the response than expected based on individual effects.

The graph of the response surface of the acid extraction yield solubilized in pepsin, evaluating the variables NaOH (*X*_1_), indicates which variables resulted in a higher yield. In Figure 2A, the yield was greater when NaOH (*X*_1_) increased and CH_3_COOH (*X*_2_) decreased the volume. In Figure 2B, the yield increases on two occasions, both when NaOH (*X*_1_) was used in the smallest volume and with stirring (*X*_3_) in the smallest agitation, and when using NaOH (*X*_1_) in the largest volume and with stirring (*X*_3_) in the greatest mechanical agitation. In Figure 2C, it is noted that the highest yield occurred when agitation with NaOH (*X*_1_) occurred, and the volume of CH_3_COOH (*X*_2_) was smaller.

**Figure 2:**
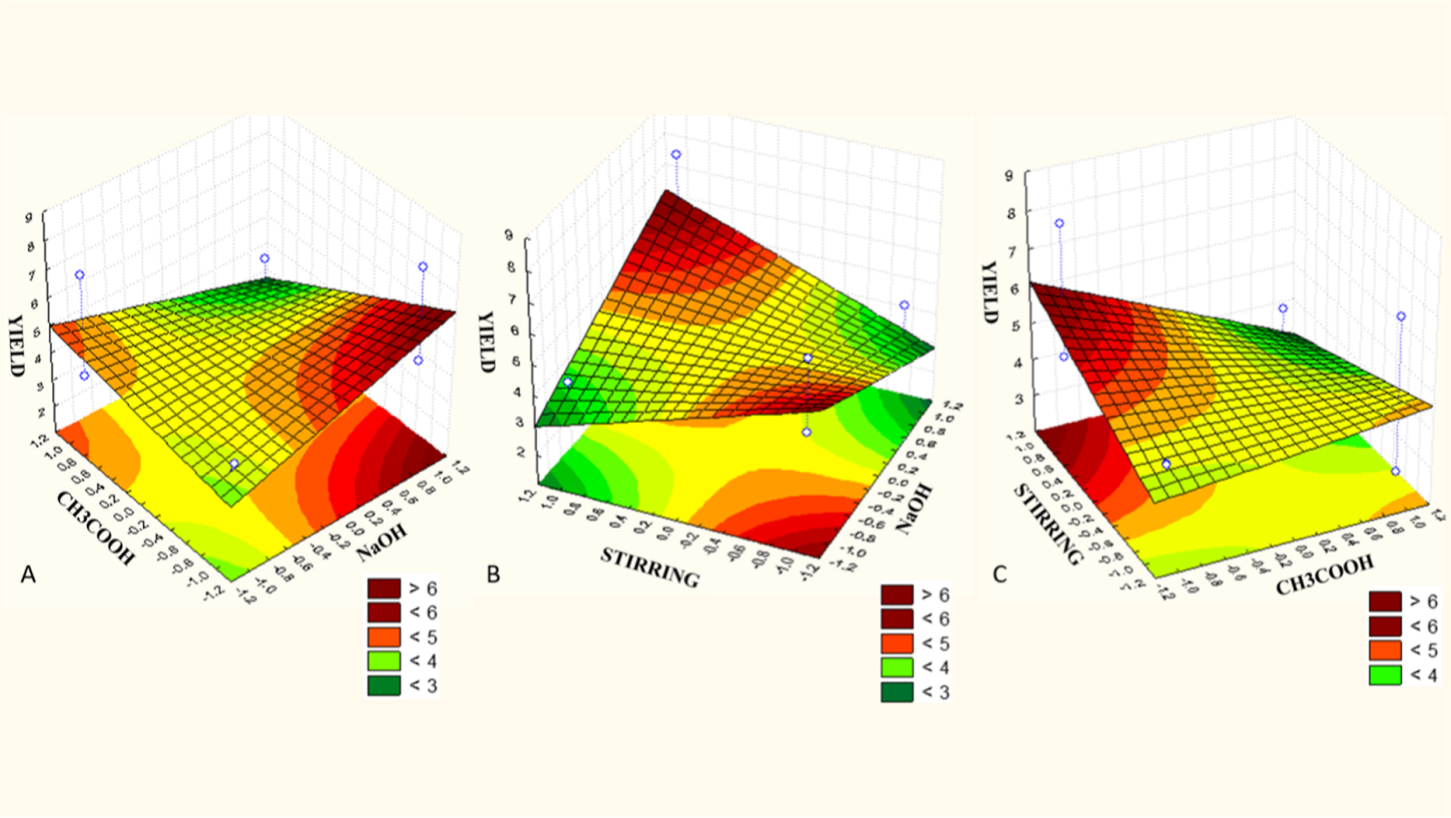
Yield response surface graphs from acid extraction with pepsin: (A) response surface of NaOH (sodium hydroxide—*X*_1_) and CH_3_COOH (acetic acid—*X*_2_); (B) response surface between mechanical stirring (*X*_3_) and sodium hydroxide (*X*_1_); (C) response surface between mechanical stirring (*X*_3_) and acetic acid (*X*_2_).

To evaluate the yield, we observed a synergistic interaction between the larger volume of CH_3_COOH (*X*_2_) and constant mechanical agitation for both extractions, resulting in a higher yield. For acid extraction, CH_3_COOH (*X*_2_), stirring (*X*_3_), and the *X*_2_ *X*_3_ interaction showed significant results (*p* < 0.05) for increased yield. On the other hand, for acid extraction with pepsin, the *X*_1_ *X*_3_ interaction was the one that presented a significant result (*p* < 0.05), showing the importance of constant mechanical agitation for increasing yield. Similar yield values were described in the literature; for Wami tilapia, the acid extraction yield ranged from 3.88–8.24%,^55^ and yield values of 4.27% for Nile tilapia were obtained.^20^ For acid extraction with pepsin, the yield of skin collagen from Nilem fish was 4.25–6.18%,^56^ and for Nile tilapia, a value of 7.60% was obtained.^20^

Different yield values in acid extraction and acid extraction with pepsin for Nile Tilapia can be found in the literature. The yield can be affected by several factors such as the extraction method, whether or not pretreatment of the skin is carried out, and the type of extraction solvent. Furthermore, the rate of addition of the pepsin enzyme concentration in collagen extraction with highly acidic solvents is dependent on the type of fish and the composition and configuration of the collagen.^57^ It is important to highlight that the size of the skin, the age of the fish, and the environment (type of water, room temperature, and feed) where the fish is raised can also affect collagen yield.

### 3.3 Characterization of Tilapia Skin Collagen

Hydroxyproline is used to indicate the presence of collagen since it is not commonly found among noncollagenous proteins; its presence stabilizes the triple helix conformation of collagen^58^ (Table 3). The collagen structure is characterized by a Gly-X-Y tripeptide repeat, where X and Y are generally proline and hydroxyproline, respectively.^59^ For both acid extraction (COL) and acid extraction with pepsin (pCOL), equivalent results (2–9 g of hydroxyproline/100 g of protein) were found in the literature.^4,20,60^ Apparently, collagen obtained from freshwater fish has greater thermal stability than marine fish, and this can be justified by the higher hydroxyproline content or even the environment in which they live. Lower hydroxyproline contents have been observed in marine fish compared to freshwater fish and mammals.^61–63^ Once the presence of hydroxyproline in the sample has been confirmed, it is possible to proceed with the characterization analyses since this evidence indicates that it is collagen.

#### 3.3.1 Collagen Denaturation Temperature

The collagen denaturation temperature (*T*_d_) was considered when the viscous or loss modulus (*Ǵ́*) crosses the elastic or storage modulus (*Ǵ*) (Figures 5 and 6). With the COL2 extract, it was not possible to perform the analysis due to its low yield. It was noticed that the rheological behavior of the solutions containing the extracts obtained by acid extraction was mostly viscoelastic, with the exception of extracts COL 4 and COL 7 (Figure 3A,B). This result may be related to the specific pretreatment conditions: COL4 was exposed to a high concentration of NaOH (20%) without agitation, which may have promoted partial degradation of the collagen structure. COL7, although with a lower concentration of NaOH (10%), was subjected to mechanical agitation, which may have interfered with the reorganization of the fibers. These structural changes resulted in less cohesive networks and, therefore, with less elastic character. As for those obtained by acid extraction with pepsin, all extracts showed elastoviscous behavior (Figure 4A,B).

**Figure 3:**
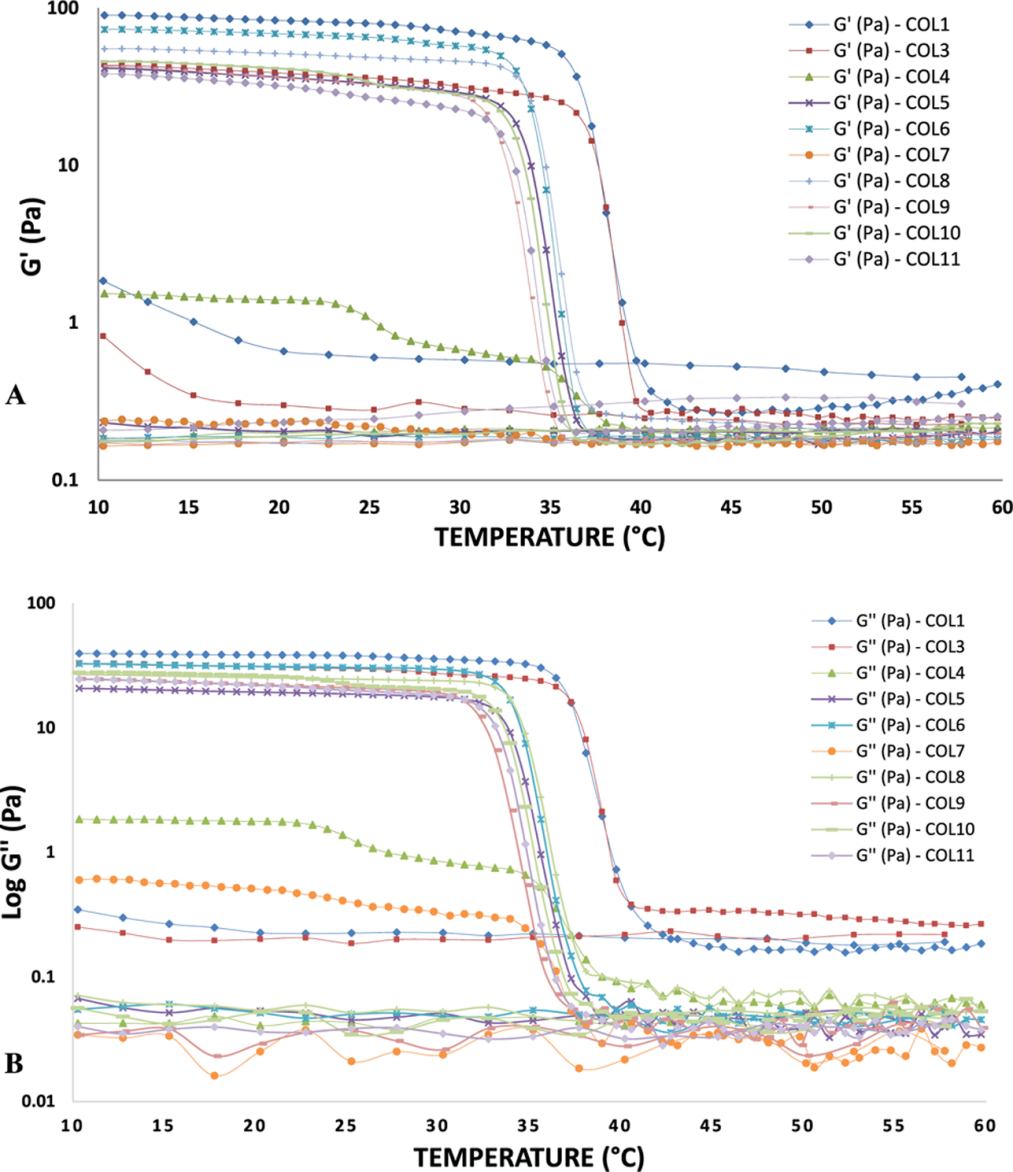
Temperature in relation to the viscous dissipation modulus (B—G″) and the elastic recovery modulus (A—G′) of acid extraction (COL).

**Figure 4:**
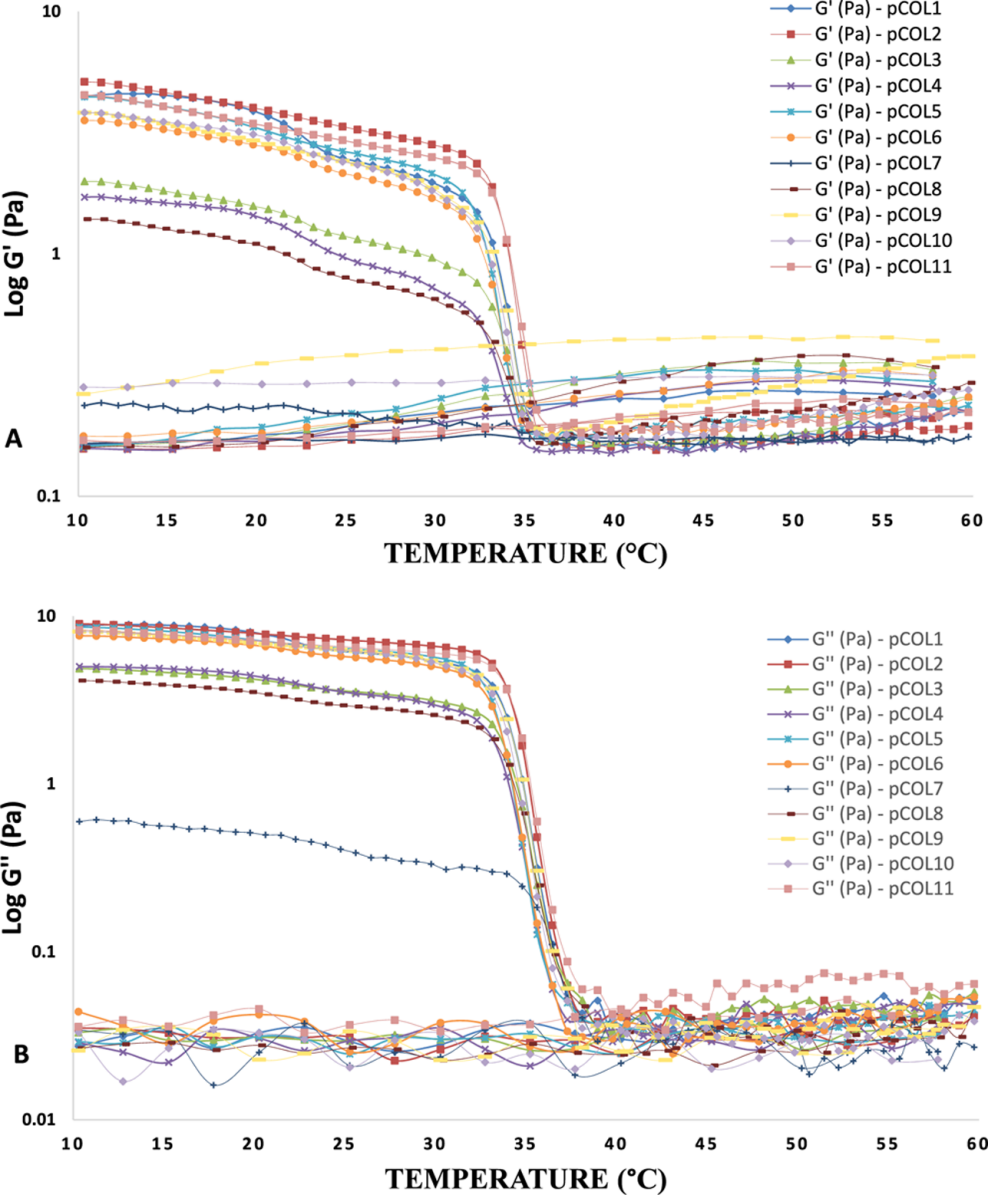
Temperature in relation to the viscous dissipation modulus (B—Ǵ́) and elastic recovery modulus (A—Ǵ) of acid extraction with pepsin (pCOL).

For both types of collagens (from methods COL and pCOL), the denaturation temperature occurred around 32.62–37.55 °C (Table 4). Equivalent results were found in other studies.^56,59^ The collagen denaturation temperature is influenced by the animal’s living environment, especially in relation to the temperature of the water where the fish lives. Collagens from freshwater fish tend to present greater thermal stability, which may be related to the greater presence of imino amino acids (proline and hydroxyproline) and the physiological adaptation of these organisms to the ambient temperature of the habitat where the animal source is located.^64^

**Table 4:** Denaturation Temperature (*T*_d_) of Tilapia Skin Extracts Obtained by Acid Extraction, without (COL) and with Pepsin (pCOL): Amount of Sodium Hydroxide (NaOH); Amount of Acetic Acid (CH_3_COOH); Mechanical Stirring (ME); with Stirring (wS) for 4 h; 1 h of Stirring and 1 h without Stirring for 4 h (wnS); No Stirring (nS)

Independent variables	Levels		
	–1	0	1
*X*_1_ = NaOH (%)	10	15	20
*X*_2_ = CH_3_COOH (%)	20	35	50
*X*_3_ = ME (type)	nS	wnS	wS

Standard run (extraction)	(%, w/w)			*T*_d_ (°C)	
	*X*_1_	*X*_2_	*X*_3_	COL	pCOL
1	–1	–1	–1	37.55	36.11
2	1	–1	–1		36.3
3	–1	1	–1	36.40	35.87
4	1	1	–1	36.47	35.48
5	–1	–1	1	34.13	35.33
6	1	–1	1	34.57	35.39
7	–1	1	1	35.55	35.87
8	1	1	1	34.9	35.97
9 (c)	0	0	0	32.65	35.98
10 (c)	0	0	0	33.31	35.7
11 (c)	0	0	0	32.62	36.35

The crossover point between *G*′ and *G*″ therefore corresponds to the transition from gel to solution, is associated with the rupture of the structural interactions responsible for maintaining the triple helix, and is widely recognized as the thermal denaturation point of collagen. It is noted that at higher temperatures, the samples underwent irreversible denaturation, characterized by the loss of structural stability and the viscoelastic behavior typical of collagen. This change in structure with *T*_d_ reflects the breakdown of the collagen triple helix into a random spiral. In other words, there is a breaking of bonds that stabilizes the secondary structure of collagen, indicating its denaturation.^57^ A *T*_d_ of collagen close to room temperature is not advisable, as this can drastically alter the physicochemical, biological, and mechanical properties of collagen, which is why its topical application is convenient since the body temperature is around 34 °C. Scientific evidence indicates that denatured collagen matrices promote more effective cellular responses than native collagen, due to the greater exposure of bioactive motifs, such as the arginine–glycine–aspartic acid sequence, which facilitates interaction with cellular receptors. Furthermore, denaturation confers greater molecular mobility and susceptibility to enzymatic degradation, characteristics that favor tissue remodeling and accelerate regenerative processes, being particularly relevant in tissue engineering and wound healing applications.^65^ Therefore, the partially denatured structure of collagen facilitates the interaction with cells, promotes adhesion and proliferation, and accelerates fundamental biological processes in healing and tissue regeneration. These factors justify the use of denatured collagen in biomedical products aimed at healing and tissue engineering.

The amounts of hydroxyproline and proline are important for the structural integrity of collagen; their higher value contributes to greater thermal stability and better rheological properties. The difference in amino acid content means that fish collagen compared to bovine collagen has poorer thermomechanical and biological properties, which limits its use in medical applications.^66^ Furthermore, compared to mammalian collagen (39–40 °C), fish collagen has a lower *T*_d_, ranging between 25 and 30 °C for most fish species, which restricts its application in biomedical technology.^67^ Although its application is limited, we cannot rule out its use, since collagens made from fish and invertebrate sources, including skin, bones, cartilage, and scales, are more bioavailable, and absorb up to 1.5 times better than bovine collagen or pork.^68^ Furthermore, studies demonstrate that the *T*_d_ of tilapia byproducts is higher than that of marine collagens, which implies an advantage for its application in the manufacture of biomedical materials.^1^

#### 3.3.2 Raman Spectroscopy

Given the results obtained so far, the chosen collagens were COL5 and pCOL6. For this choice, the extractions with the highest yield values were considered. Raman spectroscopy is a widely used technique for evaluating physicochemical and conformational changes in a wide variety of materials.^69^ In the context of TS samples, the acquisition of average spectra (four replicates) of samples COL5 (A) and pCOL6 (B) was performed at room temperature (∼23 °C) in the spectral range of 1800–600 cm^–1^. Figure 5 displays these spectra, which are used for the analysis and interpretation of the vibrational characteristics of the samples.

**Figure 5:**
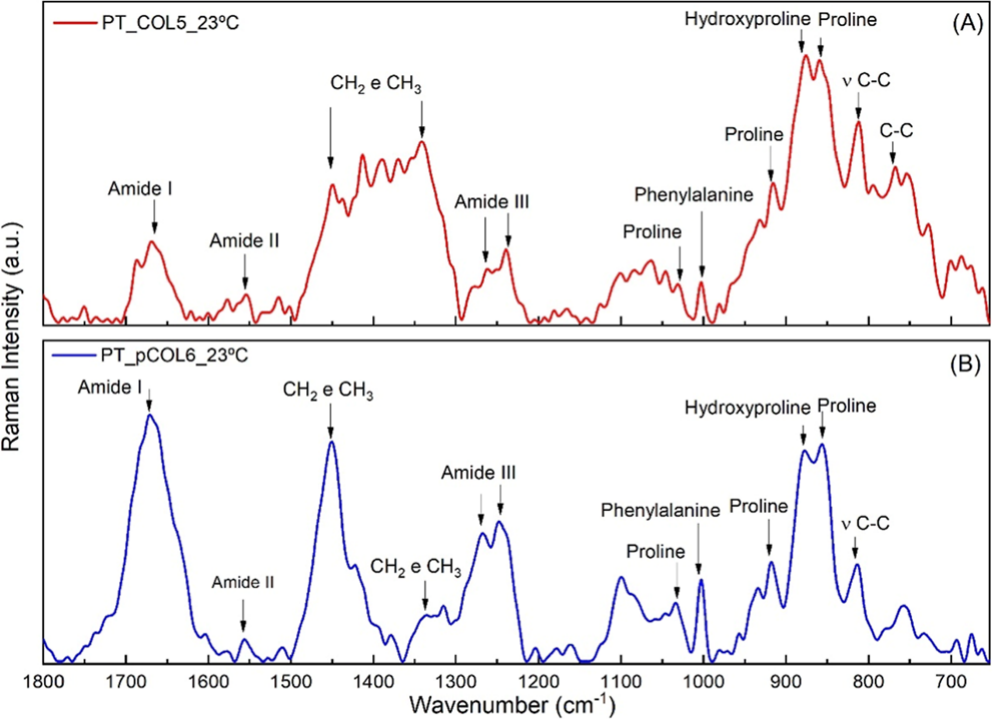
Raman spectroscopy of COL5 (A) and pCOL6 (B) tilapia skin collagen at room temperature (∼23 °C).

The band observed at 1668 cm^–1^ corresponds to amide I vibrations, which result from the stretching of C=O bonds in polypeptide chains, constituting an important indicator of the presence of proteins. The amide I spectral region is often used in the analysis of secondary structures of proteins, due to its high sensitivity to these structures.^70,71^ In relation to amide II, a band centered at 1555 cm^–1^ was observed related to the bending modes of the N–H bonds and stretching of the C–N bonds. In the amide III region, a shift in the bands was observed when comparing the two extraction methods used to prepare the samples. For sample COL5 (Figure 5A) (acid extraction), the bands are centered at 1262 and 1239 cm^–1^, while for sample pCOL6 (Figure 5B) (acid extraction with pepsin), they are centered at 1267 and 1248 cm^–1^. Importantly, the amide III region characterizes the conformational modes of protein structure.^71–73^

For the region that comprises the vibrational modes of CH_2_ and CH_3_ molecules, there was a significant difference in the shape of the bands, in relation to the extraction method. Both samples presented a band centered at 1450 cm^–1^; however, for pCOL6 (Figure 5B), a more significant contribution was observed with a peak more evident in relation to COL5 and another band centered at 1341 cm^–1^ that was more evident in sample COL5 (Figure 5A) and appeared as a secondary band in pCOL6 at the same wavenumber value. These differences may be related to the action of the acidic environment and/or the pepsin enzyme during the extraction process. During acidic extraction, the collagen fibers are solubilized and separated, forming a protein matrix enriched in collagen. In the case of acid extraction with pepsin, this enzyme promotes the specific cleavage of peptide bonds, including the removal of telopeptides, potentially altering CH_2_ and CH_3_ bond environments.^74^ The vibration mode related to the phenylalanine ring was confirmed in both samples, evidenced by the band present at 1003 cm^–1^.^75,76^

The bands present in the region between 950 and 750 cm^–1^ refer to stretching vibrations of carbon bonds ν­(C–C). In sample COL5 (Figure 5A), bands were observed at 813 cm^–1^ and 767 cm^–1^. Additionally, both samples exhibit bands near 916, 857, and 877 cm^–1^, which are attributed to the C–C stretching modes in proline and hydroxyproline residues. A weak band near 752 cm^–1^ was also observed in pCOL6.^70,76^ It is important to clarify that hydroxyproline contributes to the thermal stability of collagen through intramolecular hydrogen bonding, particularly involving the hydroxyl group of Hyp, which stabilizes the triple helix structure.^77^

The characterization of conformational changes in COL5 and pCOL6 samples as a function of temperature was also analyzed using Raman. Temperature is a determining factor that influences and modifies the structure and physicochemical properties of proteins, resulting in changes in their biological functions. In the specific case of collagen, exposure to elevated temperatures causes unfolding of the triple helix, without affecting the integrity of the primary structure;^78^ reversible conformational changes may occur, while at higher temperatures, irreversible denaturation is observed. To investigate these effects, Raman spectroscopy measurements were conducted as a function of temperature (24 °C, 34 °C, 40 °C, 50 °C, and 60 °C) on both samples.

Figure 6 shows the results for the sample COL5. In the amide III region, a band at 1244 cm^–1^ remains with an increasing temperature. A band at 1258 cm^–1^ is also present from 24 °C and exhibits changes in intensity before disappearing after 40 °C, followed by the emergence of a new band at 1275 cm^–1^. These changes are indicative of modifications in the hydrogen bonding network that stabilizes the α-helical structure, leading to reversible conformational rearrangements.^72,73,78^

**Figure 6:**
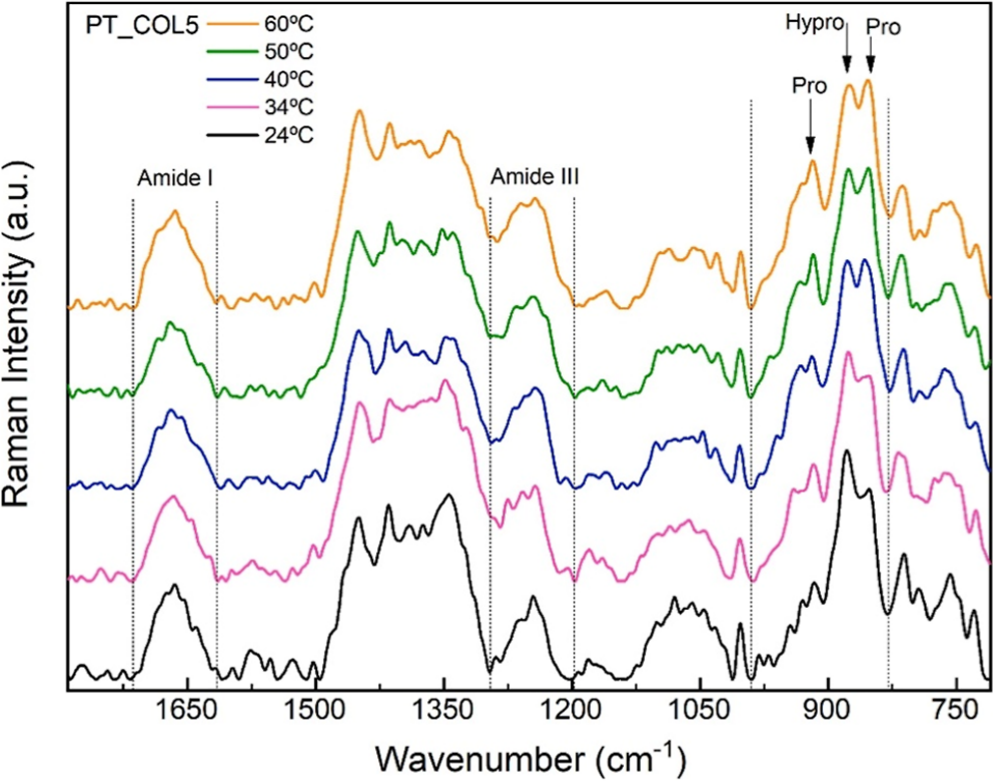
Raman spectrum of sample COL5 as a function of temperature (24, 34, 40, 50, and 60 °C).

The amide I region shows a peak at 1640 cm^–1^ at 24 °C that disappears after 34 °C. At 34 °C, new peaks appear at 1624 and 1643 cm^–1^. A secondary band at 1685 cm^–1^ arises from 40 °C onward. These shifts suggest relaxation of the triple helix structure. As seen, the region that comprises amide I is associated with the vibrational modes of stretching of C=O bonds in polypeptide chains, which are involved in protein secondary structure, in addition to being commonly used to analyze the secondary structures of proteins, which are highly sensitive.^70,71^ Thus, the observed changes may be indicative that the triple helix structure of collagen, which is supported by hydrogen bonds between the C=O carbonyl group and the glycine-NH group, has undergone relaxation.^39^

For the spectral region between 950 and 750 cm^–1^ corresponding to the vibrational stretching modes of carbon bonds ν­(C–C), observed mainly in the amino acids proline, hydroxyproline, tyrosine, and tryptophan present in the residues of the proline and hydroxyproline rings, it was found that the bands attributed to proline, as well as the band associated with hydroxyproline, remained stable throughout the heating process.^70,76^ However, it was observed that the bands centered at 930 cm^–1^ and 945 cm^–1^, clearly present at a temperature of 24 °C, diminished after 34 °C, with a transient band at 940 cm^–1^ at 34 °C which persisted only at that temperature and was no longer observed at higher temperatures. Additionally, from 40 °C onward, the emergence and gradual displacement of a secondary band at 960 cm^–1^ was observed, which moved to 968 cm^–1^ at 50 °C and to 976 cm^–1^ at 60 °C.

These results indicate specific changes in the spectral region corresponding to the vibrational stretching modes of carbon bonds present in proline and hydroxyproline residues during the heating process. While most bands associated with proline and hydroxyproline remained stable, indicating a relatively lower susceptibility of these residues to thermal disruption, the changes observed at 930 cm^–1^ and 945 cm^–1^, as well as the transient appearance of a band at 940 cm^–1^ at 34 °C, suggest localized conformational disturbances. Moreover, the progressive shift of a secondary band from 960 cm^–1^ to higher wavenumbers (up to 976 cm^–1^ at 60 °C) is consistent with ongoing conformational rearrangements and altered molecular interactions within the collagen structure during thermal treatment.^79,80^

Figure 7 illustrates the results obtained for the pCOL6 sample in relation to the temperature function. In this case, no significant changes were initially observed in comparison to those of the COL5 sample. However, closer inspection revealed changes between 1350 and 1500 cm^–1^, particularly in the intensity of CH_2_ and CH_3_ deformation bands. These variations suggest moderate conformational adjustments that may reflect subtle reorganizations within the collagen matrix. These results agree with the findings reported by Changwei Cao and collaborators, who stated that the extraction of collagen by the acid method with pepsin provides greater thermal stability, attributed to an increase in the amount of hydrogen bonds as a characteristic of the extraction method.^74^

**Figure 7:**
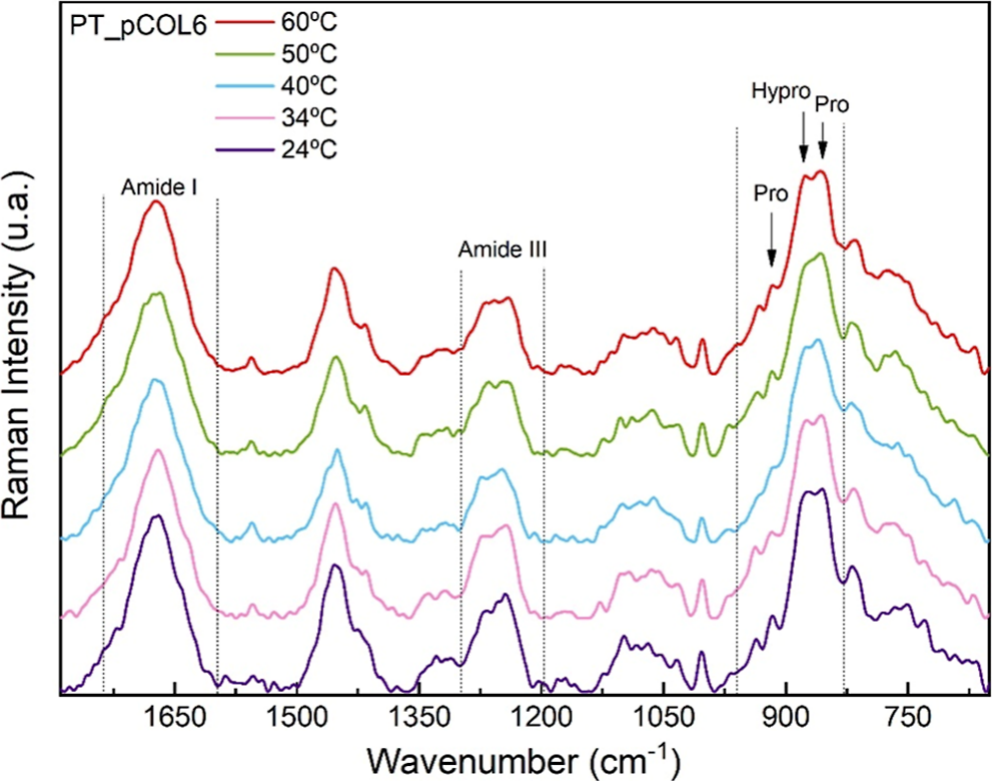
Raman spectrum of the pCOL6 sample as a function of temperature (24, 34, 40, 50, and 60 °C).

Raman spectroscopy analysis as a function of temperature confirmed that the collagen extracted with pepsin (pCOL6) demonstrated greater thermal stability when compared to that of the collagen obtained only by acid extraction (COL5). Both samples showed typical collagen bands at room temperature; however, with an increase in temperature, differences in structural behavior were observed. In the COL5 sample, changes in the amide I and III regions, as well as in bands related to proline and hydroxyproline, indicated that conformational rearrangements may start around 34 °C, suggesting partial loss of the triple helix structure. On the other hand, the pCOL6 sample showed more stable spectral features throughout the heating process, with only moderate variations, especially between 1350 and 1500 cm^–1^, without robust evidence of denaturation. These results suggest that the use of pepsin during the extraction enhances the structural organization of collagen, probably due to the removal of telopeptides, and helps to maintain the integrity of the triple helix when the temperature increases. In general, the spectral changes observed in pCOL6 indicate reversible conformational changes up to 60 °C, supporting the structural preservation of the collagen molecule under thermal conditions.

#### 3.3.3 Thermal Analysis

DSC analyses were carried out to evaluate the effect of the temperature on the two types of extractions (COL5 and pCOL6). The results show that there was only one peak within the temperature range of 20 to 60 °C for both COL5 and pCOL6 (Figure 8), which may indicate thermal denaturation and water bound between the collagen molecules. COL5 collagen showed a transition curve at a maximum temperature (*T*_max_) of 35.88 °C with denaturation beginning at 32.74 °C. However, pCOL6 collagen showed a *T*_max_ transition curve of 36.77 °C, with denaturation beginning at 33.38 °C. Similar results were found in other studies, with the denaturation temperature varying between 32 and 37 °C in collagen samples obtained from fish skin.^8,56,81,82^

**Figure 8:**
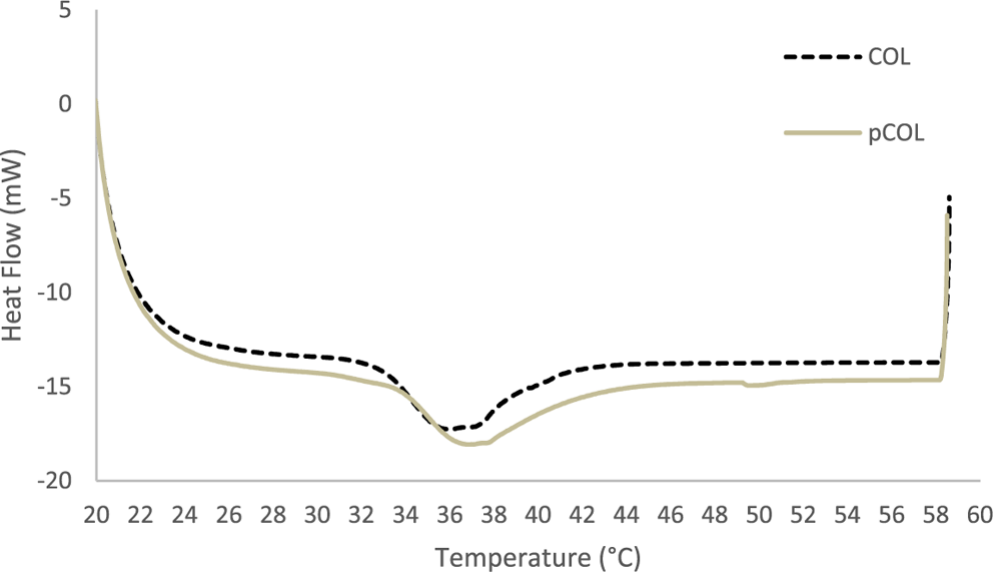
Differential scanning calorimetry (DSC) curves represent the heat flow evolution of collagen (COL and pCOL) with increasing temperature.

Although both collagens (COL5 and pCOL6) belong to type I collagen and have a complete triple helix structure, there are still some differences in thermal stability, which can be attributed to the removal of telopeptides.^8^ Furthermore, collagen’s tripeptide chains are kept linked by noncovalent bonds, such as hydrogen bonds, which are essential for their stability. When collagen molecules undergo thermal variation, these noncovalent bonds are broken, causing the transformation of the triple helix structure into a random coil structure, which changes the properties of the collagen.^2^

The TGA of both collagens (COL5 and pCOL6) analyzed showed two stages of mass loss (%) shown in Figure 9A. In the first stage, COL5 and pCOL6 lose 13.59% (in the range of 28–161.95 °C) and 11.37% (40.00–170.00 °C) of mass, respectively. In the second stage, COL5 loses 63.37% (165.00–322.50 °C) of mass and pCOL6 loses 65.11% (170.00–322.00 °C). The first stage can be attributed to the loss of structural bound water, followed by thermal destruction of the polypeptide chain (collagen degradation) in the second stage.^83,84^ Similar results were found for tilapia collagen, both for weight variation and temperature variation in the two stages verified.^84–87^ In collagen extracted from Tilapia bones (*Oreochromis mossambicus*), there was also mass loss in two stages, the first notable weight loss occurred at approximately 90 °C, where the authors associated it with the removal of physically absorbed water, and the second stage, corresponding to collagen decomposition, was observed at approximately 350 °C, similar to that found in collagen obtained from TS (*O. niloticus*).^84,87,88^ During TGA, the mass of the sample is monitored while the temperature increases at a controlled rate. The resulting curve (TGA thermogram) shows the residual mass of the sample as a function of temperature. T5% is the temperature at which the residual mass is 95% of the initial mass of the sample; for COL5, the temperature was 49.66 °C, while for pCOL6, it was 63.45 °C, indicating the greater thermal stability of collagen extracted with pepsin (Figure 9A).

**Figure 9:**
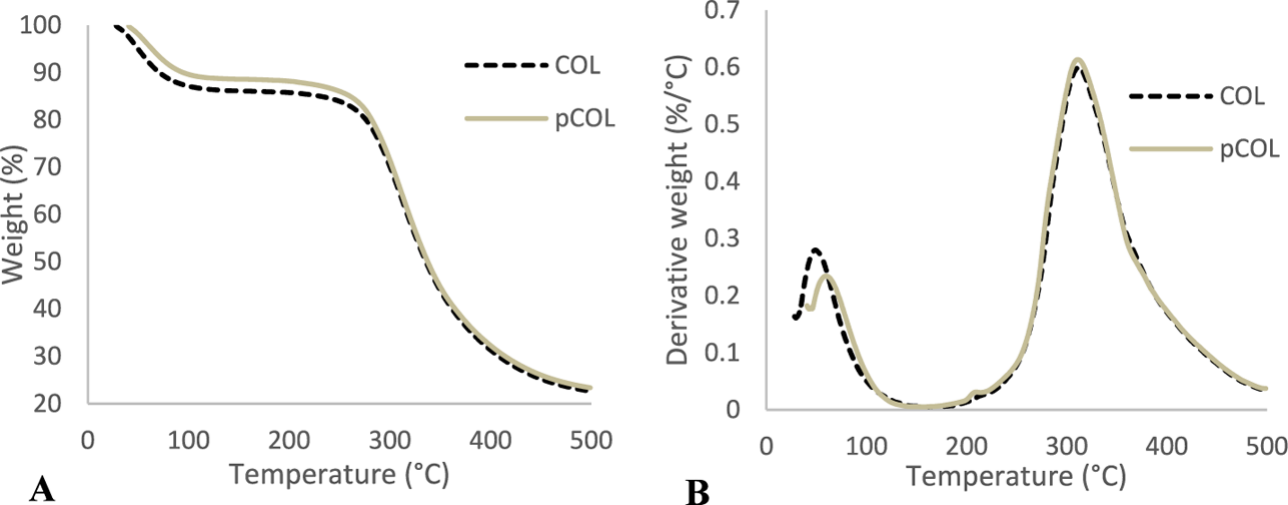
Thermogravimetric analysis (TGA) (A) and thermal glass transition temperature (DTG) (B) curves of the collagen from tilapia skin.

To enhance sensitivity in detecting minor changes in the percentage of weight loss of samples relative to temperature, a derivative graph from the thermogravimetric curve was generated.^66^ Peaks in the differential thermogravimetric analysis curve correspond to the maximum in the rate of mass loss, indicating specific decomposition events. This can help to more clearly identify distinct stages of decomposition or reactions that occur at different temperatures. As can be seen in Figure 9B, there are peaks where mass loss is faster, corresponding to specific temperatures where decomposition occurs faster. Collagen weight loss occurs rapidly with increasing temperature, especially in the range between 40 to 100 °C and 200 and 400 °C (Figure 9B). The first thermal peak resembles the denaturation of collagen and evaporation of unbound water, being 48.91 °C for COL5 and 62.58 °C for pCOL6. The second exothermic peak displays a maximum temperature of 311.71 °C for COL and 310.54 °C for pCOL, which is associated with collagen degradation and evaporation of the residual group.

#### 3.3.4 X-ray Diffraction

The XRD patterns of the COL and pCOL samples are presented in Figure 10. Both samples exhibited two well-defined diffraction angles (2θ) characteristic of type I collagen, located at 2θ of 7.50° and 21.20° for the pCOL sample and 7.40° and 20.20° for the COL sample, respectively. The first diffraction peak, which is more intense and at a smaller angle (∼7°), is associated with the periodic spacing between the molecular chains of the collagen triple helix structure. The second peak, wider and at an intermediate angle (∼20°), is related to the lateral packing of the collagen fibrils.^89,90^ From the Bragg equation (*d* = λ/2sinθ), the interplanar spacings (*d*) were calculated, resulting in 11.77 and 4.19 Å for the pCOL sample and 11.93 and 4.39 Å for the COL sample. These d values indicate the presence of a molecular organization compatible with the native structure of type I collagen. The maintenance of these characteristic peaks suggests that the supramolecular structure of collagen was preserved during the extraction processes used in this study, indicating that COL and extracted COL were in their native conformations and not denatured.^90^ The structural integrity demonstrated is a positive indication for possible pharmaceutical and biomedical applications since the biological activity and functionality of collagen are directly related to the preservation of its conformation.

**Figure 10:**
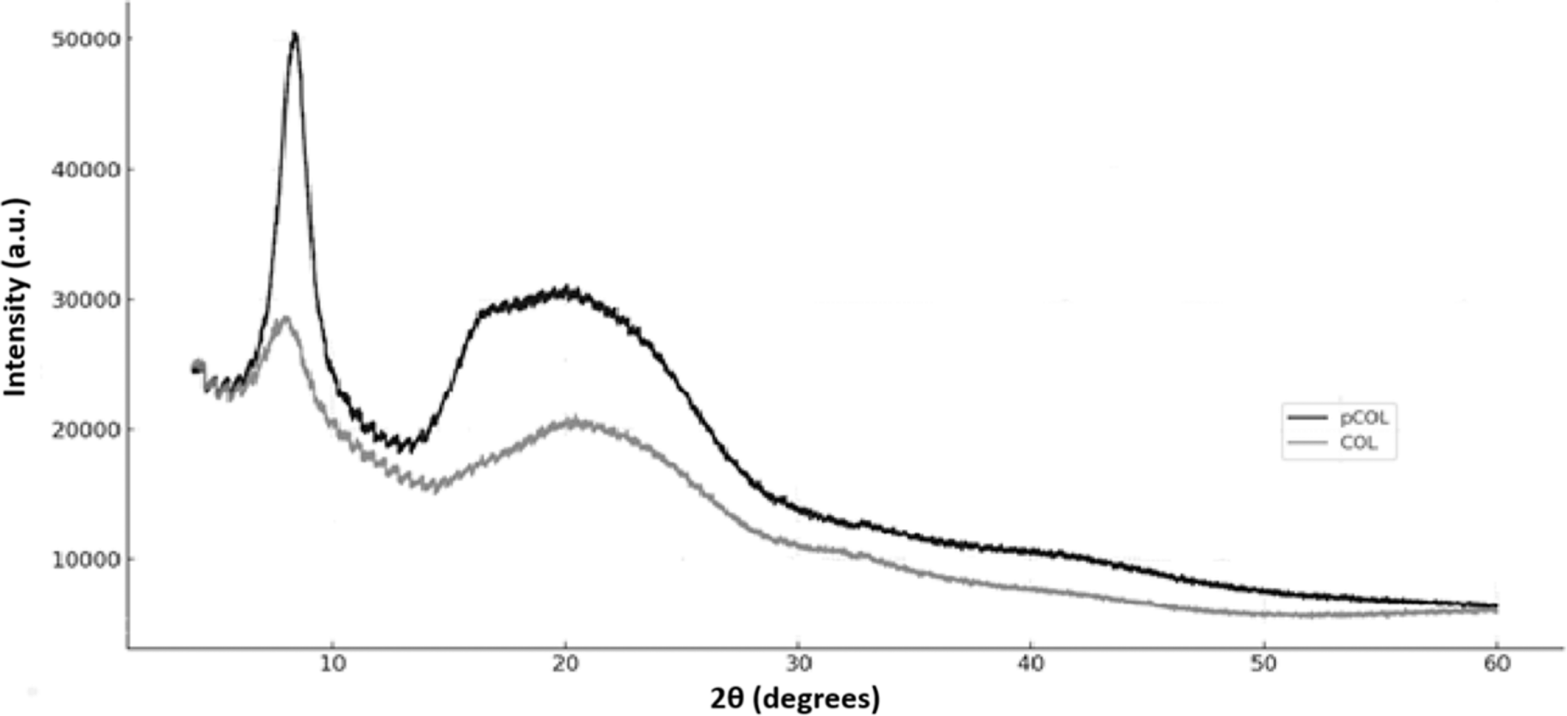
X-ray diffraction (XRD) patterns of collagen COL and pCOL observed at temperatures from 4° to 60°.

#### 3.3.5 Circular Dichroism

CD spectroscopy typically provides highly sensitive measurements, allowing for the use of low sample volumes and concentrations. At temperatures below 36 °C, positive peaks for the triple helix were observed at 222 nm (Figure 11). By fixing the ellipticity at 222 nm (since changes in the positive band at this wavelength are sensitive to the structure of the triple helix) and analyzing it at different temperatures, it is possible to notice that from a temperature of 32 °C, thermal denaturation occurs, due to the fact that the positive peak begins to disappear and the intensity of the negative peak decreases.^44,91^ The presence of pepsin (pCOL6) can remove parts of the collagen structure, such as telopeptides, which are nonhelical regions at the ends of collagen molecules. These structural changes, in the secondary structure of the protein, can affect the characteristics observed in CD spectroscopy such as the magnitude and position of absorption peaks.

**Figure 11:**
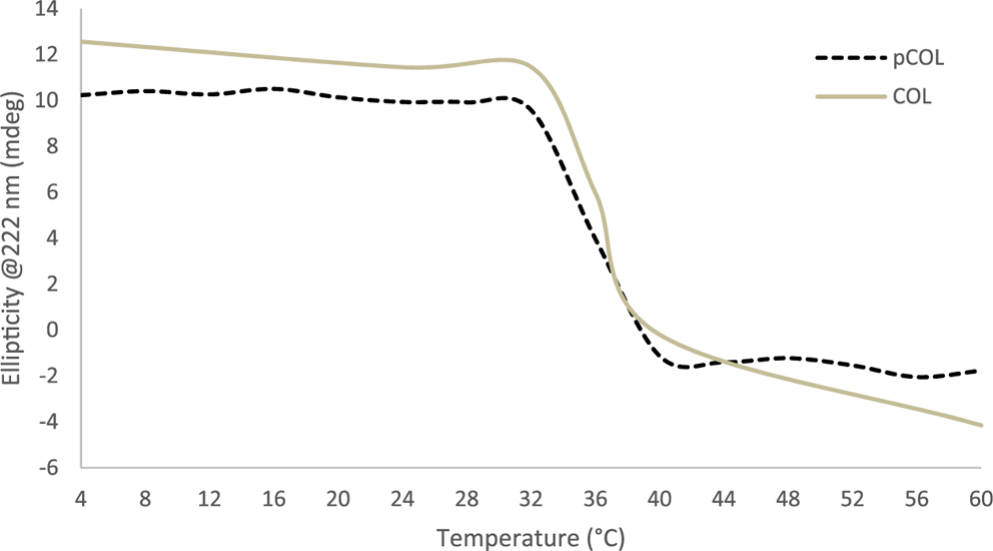
Ellipticity at 222 nm of collagen COL and pCOL observed at temperatures from 4 to 60 °C.

CD is a valuable spectroscopic tool that can be used to determine optical isomerism, identifying whether the triple helical structure is intact, and determine the secondary structure of macromolecules, as it gives rise to specific patterns and peaks according to the presence of alpha regions, helixes, β sheets/turn, and/or random coils. The CD spectra of COL5 and pCOL6 collagens were analyzed by varying ellipticity versus wavelength and ellipticity versus temperature (Figure 12).

**Figure 12:**
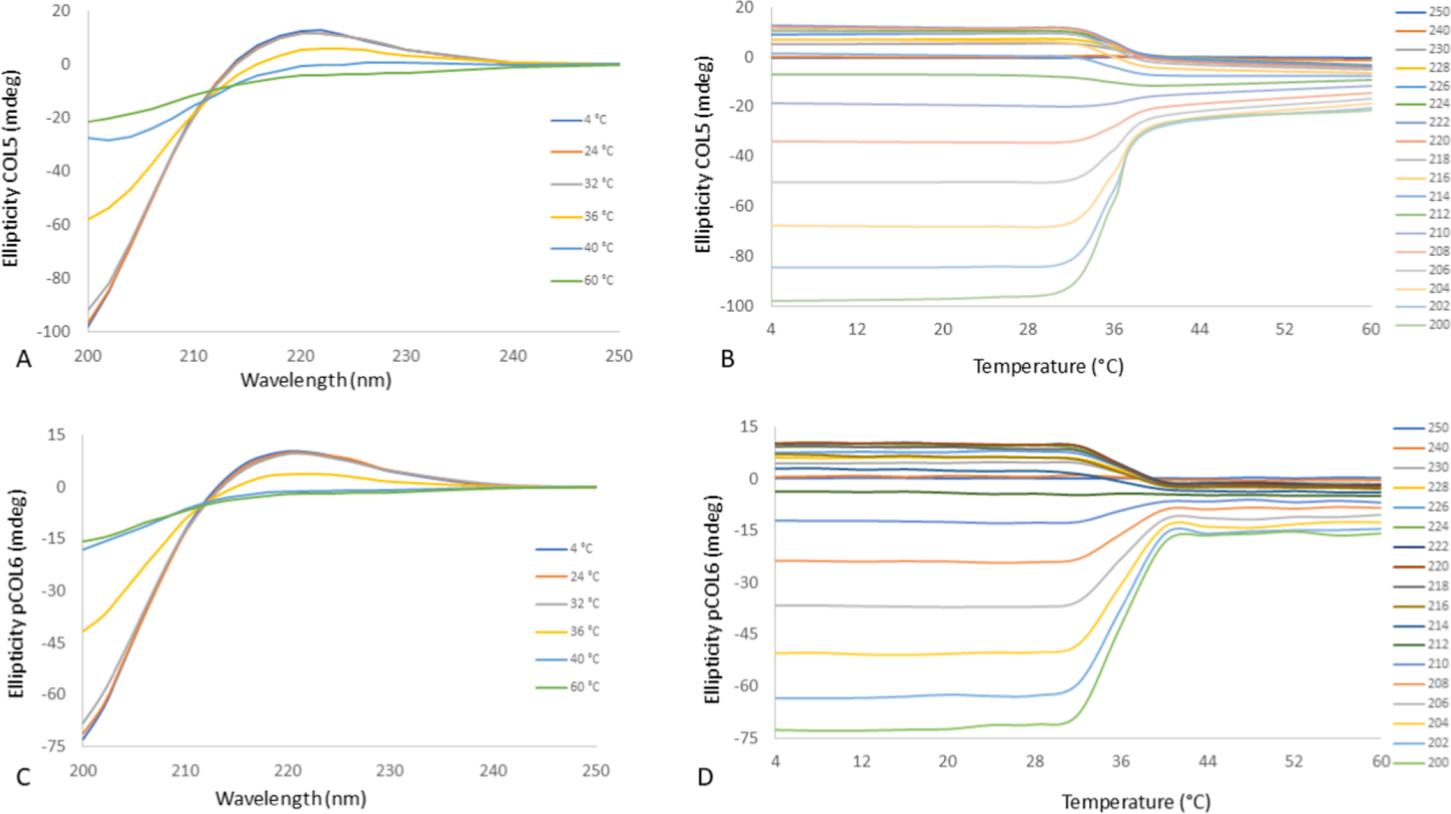
Ellipticity for collagen COL and pCOL plotted in relation to the wavelength (A,C, respectively) and in relation to temperature (B,D, respectively).

Collagen is a structural protein where three polypeptide chains, rich in glycine and proline, form a triple helix. Each of the three individual chains adopts a polypropylene II (PPII) conformation. Its presence indicates the presence of collagen in a sample. Furthermore, this triple helical structure gives collagen its strength and mechanical resistance. In the far ultraviolet region, the CD spectra of COL5 and pCOL6 collagens adopt a helical conformation similar to PPII with a positive peak between 221 and 222 nm (maximum positive Cotton effect), a negative peak at 200 nm (maximum negative Cotton effect), and a crossing point (zero rotation) between 213 and 216 nm, depending on the temperature analyzed, presenting values typical of a native collagen^90,92^ (Figure 12A,C). Between temperatures of 32 and 36 °C, the negative peak at 200 nm and the positive peak at 222 nm became more attenuated, indicating the beginning of the collagen protein denaturation process.

From a temperature of 40 °C, both types of collagen extraction showed negative ellipticity (millimeter deviation) values (Figure 12A,C), which indicates the denaturation of the protein and possibly the transition of collagen into gelatin. Unlike triple helices (which have a negative peak around 190 nm and a positive peak around 222 nm), random coils do not have this double peak structure,^44^ indicating that, under certain conditions (e.g., thermal denaturation), collagen regions can unwind and adopt random coil conformations. As the temperature increased, significant changes in spectral shape occurred between the triple helix spectra at low temperatures and the random coil spectra at elevated temperatures, indicating the loss of a stable secondary structure.

Collagen fibrillation is influenced by pH due to the surface charge of the collagen triple helix monomers, which oscillate according to pH. At very low or very high pH, a repulsive force is generated between collagen molecules, which prevents their assembly into fibrils. However, at values close to neutral pH, the net charge of the triple helices is close to zero, reducing the repulsive force and favoring the formation of fibrils.^93,94^ Collagen fibrillogenesis is favored at a pH close to neutral to slightly alkaline (pH 7.0–8.0). For this reason, for CD analysis, collagen was prepared in a PBS solution (pH = 7.3). Observing the occurrence of fibrillogenesis is important, as it involves a transition of soluble collagen into organized forms, such as fibrils and fibers, which are essential for the formation of strong and functional connective tissues. Both types of collagens do not appear to affect collagen’s ability to form fibrils. An increase or maintenance of positive ellipticity was observed around 220–230 nm, indicating an ordered structure and the formation of fibrils, up to a temperature of 28 °C (Figure 12B,D).

#### 3.3.6 Scanning Electron Microscopy

The micrographs of COL5 and pCOL6 magnified 200× and 500× show slightly different morphologies (Figure 13). In the micrograph magnified at 200×, in both COL5 and pCOL6 samples, the collagen presented porosity and a fibrous and irregularly filamentous structure. Although the differences are visually discrete, COL5 exhibited a more organized and homogeneous microstructure, characterized by a reticulated arrangement of fissures and voids, and a lower density of fibrillar elements. In contrast, pCOL6 exhibited a more heterogeneous morphology, with a higher density of fibrillar elements and greater interconnectivity, suggesting better molecular self-assembly and fibrillogenesis facilitated by pepsin treatment. These results were like other studies and suggest the use of collagen, obtained from fish skin, in the biomedical area as a good drug carrier due to its fibrous structure.^2,95,96^ The microstructure presented by the two types of collagen extraction from TS (porosity, interconnectivity, and surface area) is widely recognized as an important parameter for a biomaterial with the aim of understanding its use in the biomedical field.^95^

**Figure 13:**
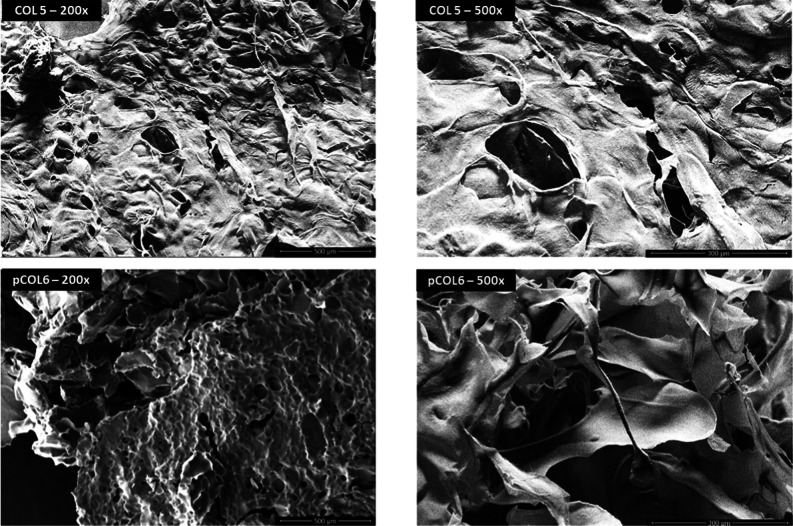
Micrographs obtained by scanning electron microscopy (SEM) of freeze-dried collagen samples, obtained by acid extraction (COL5) or acid extraction with pepsin (pCOL6), at magnifications of 200 and 500×.

This difference between COL5 and pCOL6 may have been caused by the influence of pepsin, which removes telopeptides, forming non-cross-linked collagen fibers, consistent with the results obtained in Raman spectroscopy. The complex collagen fibril form has been associated with a high water retention capacity.^72^ This ability to retain water gives collagen a beneficial characteristic for its use as a moisturizing agent in cosmetics. Furthermore, the microstructures formed by porosity and interconnectivity provide ideal physicochemical properties and biofunctionality, serving as absorption sponges and matrices to enable cell proliferation.^6,34,73^ These structural characteristics of collagen provide advantages in its use in promoting wound healing, by allowing cell migration and proliferation, indicating that both types of extraction provide collagen capable of being applied in different fields in the pharmaceutical, cosmetic, and biomedical industries.

## 4 Conclusion

The acid extraction method proved effective in obtaining collagen, in both the presence and absence of pepsin, with the cleaning phase being crucial for ensuring the purity of the collagen. The proximate composition of the byproduct (raw material) revealed lipid, protein, moisture, and ash values like those found in the literature, indicating the quality of the extracted collagen, which can be used as a pharmaceutical ingredient.

When the two extraction methods were compared, it was found that the acid extraction (COL) yielded a higher output than the acid extraction with pepsin (pCOL), although both exhibited yields close to 10%, as described in the literature. The detection of hydroxyproline in both samples provides strong evidence of the presence of collagen, given that hydroxyproline is a major amino acid found in collagenous proteins. However, additional analyses were used to confirm the identity and integrity of the collagen.

In all extractions, it was determined that the *T*_d_ of the collagen was above 32 °C, which is favorable for biomedical and pharmaceutical applications as it ensures the presence of collagen in the formulation and reduces the risks of denaturation during storage. Comparable results were found in the analyses of Raman spectroscopy, DSC, TGA, and circular dichroism. Based on the obtained data, the COL5 and pCOL6 extractions were selected for subsequent analyses.

Raman spectroscopy confirmed the presence of hydroxyproline and amides I, II, and III, which are characteristic of collagen protein. It was noted that pCOL6 extraction was more thermally stable than COL5, probably due to the cleavage of telopeptides by pepsin. XRD analysis demonstrated that the supramolecular structure characteristic of type I collagen was preserved in the COL and pCOL samples, suggesting that the extraction processes did not compromise the native conformation of the molecule.

Despite the structural differences between COL5 and pCOL6 observed in the SEM images, both samples exhibited characteristics that suggest the presence of collagen fibrils, essential for water retention and structuring, which favors cell migration and proliferation. These characteristics indicate the potential of collagen extracted from the byproduct (TS) for applications in various fields, including the pharmaceutical, cosmetic, and biomedical industries.

## Supplementary Material



## Notes

Analysis of the protection model terms related to Tilapia skin extract extraction, focusing on different parameters such as yield and hydroxyproline content in both acid extraction and pepsin extraction and providing details on the mathematical models used in each extraction method (PDF)

## Abbreviations

TS: Tilapia skin
COL: acidic extraction
pCOL: acidic extraction using pepsin
*T*_d_: denaturation temperature
DSC: differential scanning calorimetry
TGA: thermogravimetric analysis
CD: circular dichroism
SEM: scanning electron microscopy.
