# Supplementary material for: Effect of the Extraction Methods on the Physicochemical Characteristics of Collagen Derived from Tilapia (Oreochromis niloticus) Skin
Source: ACS Omega. 2025 Aug 7;10(32):35809–26. doi: 10.1021/acsomega.5c02637 (PMC12368733; doi:10.1021/acsomega.5c02637)
Supplement: Supplementary file 1 [file ao5c02637_si_001.pdf]

Effect of the Extraction Methods on the Physicochemical Characteristics of Collagen Derived from Tilapia (*Oreochromis niloticus*) Skin

Denise Tiemi Uchida[a], *Adriane do Nascimento Volnistem*[b], *Michael Thomas Cook*[c], *Marcos Luciano Bruschi*[a,*]

[a]Laboratory of Research and Development of Drug Delivery Systems, Postgraduate Program in Pharmaceutical Sciences, Department of Pharmacy, State University of Maringa, Colombo Avenue, 5790, K68, Rooms 210-214, 87020-900, Maringa, PR, Brazil

[b]Department of Physics, State University of Maringa, Colombo Avenue, 5790, 87020-900, Maringa, PR, Brazil

[c]UCL School of Pharmacy, University College London, 29-39 Brunswick Square, London, WC1N 1AX, England, United Kingdom

* Corresponding author e-mail address: mlbruschi@uem.br

Supporting Information

Table S1 – Comparison between extraction methods and different collagen sources

Source	Pre-treatment	Extraction Method	Animal
Skin	Ethanol + Sodium chloride	Acetic acid + ultrasonication	Flatfish[13]
	Sodium hydroxide + Butanol	Acetic acid	Sole fish skin (marine waste)[14]
	-	Pepsin or ethylene diamine	Chicken[15]
	Chemically pure sodium hydroxide to sodium chloride	Acetic acid	Pig carcasses[16]
	-	Eutectic solvent composed of citric acid:xylitol:water	Blue Shark[17]
	-	Acetic acid	Blue Shark[17]
	Acetic Acid	Acetic acid + Pepsin	Rabbit[18]
	Sodium hydroxide + Butyl alcohol	Acetic Acid + Collagenase by two bacteria	Salmon[19]
	Sodium hydroxide + Butyl alcohol (Chemical pretreatment) + *Bacillus velezensis* FEL-BM21 (fermentation pretreatment)	Acetic acid	Tilapia fish[20]
	Sodium hydroxide + Butyl alcohol (Chemical pretreatment) + *Bacillus velezensis* FEL-BM21 (fermentation pretreatment)	Acetic acid + Pepsin	Tilapia fish[20]
Scales	-	Acetic acid + The scales were then supplied with O2, CO2, or O3 in the form of ultrafine bubbles at a gas flow rate of 3 L/min for 6 h + Ozone cube	Tilapia[21]
	Sodium hydroxide	Double-distilled water (ddH2O), 1.26% citric acid (pH 2), or 9.37% acetic acid (pH 2) at a ratio of 4.7:1 (w/v)	Tilapia[22]
	EDTA + Sodium chloride	Pepsin + Acetic acid + Ultrasonic extraction	Carp[23]
	Calcium carbonate + Hydrochloric acid + Sodium hydroxide	Heating (80 °C) until a thick viscous fluid was formed. The viscous fluid was then spread on a flat polypropylene surface and allowed to air dry at room temperature (19 ± 5 °C).	Croaker fish (*Pseudotolitus elongatus*)[24]
Tissue	Sodium hydroxide	Acetic acid	Jellyfish[25]
Short tendons	Sodium hydroxide + Acetone	Pepsin + Acetic acid	Cattle[26]
	Sodium hydroxide + Acetone	Pepsin + Ultrasonic treatment and acetic acid solution	Cattle[26]
Mussel byssus	Sodium hydroxide + Hydrochloric acid	Pepsin + acetic acid	Mussel[27]
Leather	Sodium chloride	Perchloric acid + Acetic acid	Lime fleshings from hides (Bovine)[28]
	-	Trypsin + Acetic Acid	Raw sheepskin[29]
Pericar-dium	Sodium hydroxide + Ultra-Turrax	Pepsin + Hydrochloric acid	Bovine[30]
Feet	Sodium hydroxide	Acetic acid	Chicken[31]

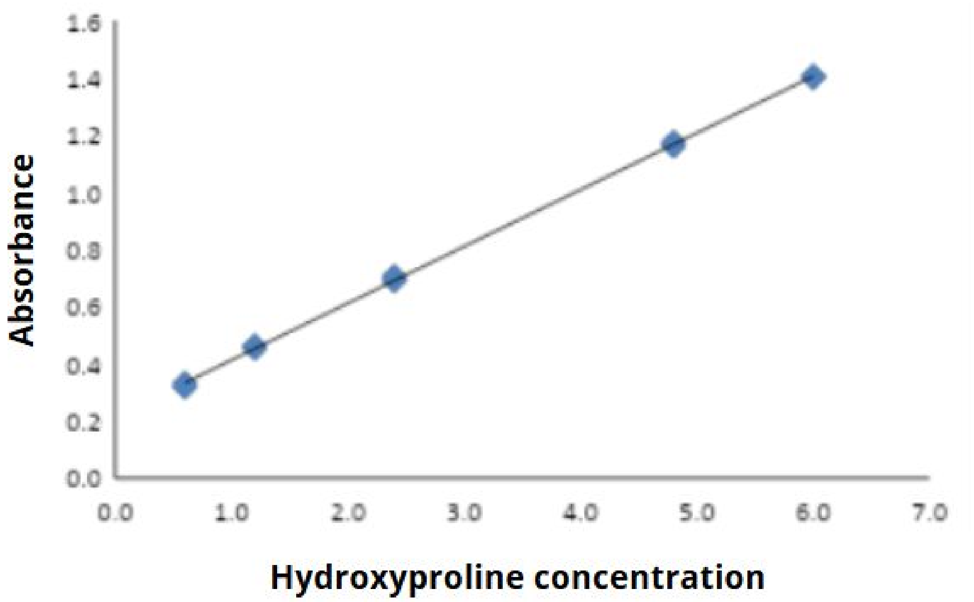

Figure S1. Standard curve of the hydroxyproline obtained by ultraviolet spectrophotometry.

The linear regression of the equation of the line was y = 0.1983x + 0.2142, showing linearity with a coefficient of determination of 0.9996 at concentrations of 0.6; 1.2; 2.4; 4.8 and 6.0 µg/mL.

Table S2. Results of the one-way analysis of variance (ANOVA) for the significance of the regression and deviation from the linearity of the analytical curve of the hydroxyproline standard

	**df**	**SS**	**MS**	**F**	***p***	**Tabulated F**
Regression	1	2.549218	2.549218	34723.41	1.15E-23	4.67
Residual	13	0.000954	7.34E-05			
Lack of Fit	3	0.000460	0.000153	3.097896		3.71
Pure Error	10	0.000495	0.000049			
Total	14	2.550172				

Note: df = degrees of freedom; SS = sum of squares; MS = mean square; F = analysis statistic; p = significance.

Residual analysis showed that the regression was significant, as the MQreg/MQres ratio (34,723.41) was much greater than the tabulated F value (4.67). Furthermore, the method did not present a lack of fit, since the MQlof/MQpure ratio (3.09) was lower than the tabulated F value (3.71) (Table S2). The limit of detection (LOD), defined as the lowest concentration of the substance that can be detected, was 0.05875 µg/mL. Meanwhile, the limit of quantification (LOQ), which is the lowest amount of hydroxyproline that can be quantitatively determined, was 0.195858 $\mu g.mL^{-1}$. The data obtained showed a relative standard deviation (RSD) lower than 5%.

Table S3. Equation model terms on tilapia skin extraction yield

Terms	Coefficient	Standart error	p – value
Degree of curvature	4.10	0.33	0.0011
X_1	-1.67	0.78	0.1199
X_2	-3.95	0.78	0.0147
X_3	4.07	0.78	0.0135
X_1X_2	1.33	0.78	0.1851
X_1X_3	0.77	0.78	0.3928
X_2X_3	-3.97	0.78	0.0144
$X_1X_2X_3$	1.07	0.78	0.2603

X_1 = Volume of NaOH; X_2 = Volume of acetic acid and X_3 = Mechanical stirring.

The p-values less than 0.05 categorize the significant terms in the model. All terms in the model were used to generate the equation that best justifies the mathematical model (Equation 1):

$$y = 4.10 - 3.95X_2 + 4.07X_3 - 3.97X_2X_3 \quad (1)$$

Table S4. Equation model terms on the extraction yield of tilapia skin extract solubilized in pepsin

Terms	Coefficient	Standart error	p – value
Degree of curvature	4.44	0.30	0.0001
X_1	0.04	0.35	0.9102
X_2	-0.51	0.35	0.2130
X_3	0.097	0.35	0.7934
X_1X_2	-0.84	0.35	0.0714
X_1X_3	1.08	0.35	0.0364
X_2X_3	-0.65	0.35	0.1329
$X_1X_2X_3$	4.44	0.30	0.0001

X_1 = Volume of NaOH; X_2 = Volume of acetic acid and X_3 = Mechanical stirring.

All terms were used to generate the equation that best justifies the mathematical model (Equation 2):

$$y = 4.44 + 1.08X_1X_3 \quad (2)$$
